# The Influence of Certain Operating Conditions of the FDM Process on the Mechanical Properties of Polymeric Materials—A Review

**DOI:** 10.3390/polym18101183

**Published:** 2026-05-12

**Authors:** Vlada Țisari, Marius Andrei Mihalache, Gheorghe Nagîț, Vasile Ermolai, Alexandru-Ionuț Irimia, Cosmin-Gabriel Grădinaru, Alexandra-Anamaria Spiridon, Elisaveta Crăciun, Roxana-Gabriela Hobjâlă, Laurențiu Slătineanu

**Affiliations:** 1 Department of Machine Manufacturing Technology, “Gheorghe Asachi” Technical University of Iasi, 700050 Iași, Romania; vlada.tisari@student.tuiasi.ro (V.Ț.); marius-andrei.mihalache@academic.tuiasi.ro (M.A.M.); gheorghe.nagit@academic.tuiasi.ro (G.N.); alexandru-ionut.irimia@academic.tuiasi.ro (A.-I.I.); cosmin-gabriel.gradinaru@student.tuiasi.ro (C.-G.G.); elisaveta.craciun@student.tuiasi.ro (E.C.); roxana-gabriela.hobjala@student.tuiasi.ro (R.-G.H.); laurentiu.slatineanu@academic.tuiasi.ro (L.S.); 2 Department of Digital Production Systems, “Gheorghe Asachi” Technical University of Iasi, 700050 Iași, Romania; alexandra-anamaria.spiridon@student.tuiasi.ro

**Keywords:** fused deposition modeling, polymeric material, mechanical properties, influencing factors, tensile strength, bending strength, hardness, modeling, optimization

## Abstract

The use of parts made of polymeric materials has occasionally highlighted the need for them to possess the best possible mechanical properties. One of the currently widely used processes for manufacturing parts from polymeric materials is fused deposition modeling. This process allows for variations in the magnitudes defining the mechanical properties of polymeric materials to be obtained through an appropriate selection of the process input factor values. The analysis of the process has highlighted the primary factors capable of affecting the values of parameters corresponding to the mechanical properties of polymeric materials. The opinions formulated by various researchers regarding the influence of fused deposition modeling application conditions on some of the mechanical properties of polymeric materials have been synthetically and systematically presented. In terms of mechanical properties, tensile strength, compression strength, elongation at break, flexural strength, torsional strength, impact strength, fatigue resistance, and hardness were taken into consideration. Some modeling and optimization solutions for the influence exerted by the 3D printing process input factors on the values of the parameters defining the mechanical properties of polymeric materials in parts manufactured via the FDM process were also highlighted.

## 1. Introduction

Due to the broad scope of 3D printing technology, multiple definitions and concepts are introduced in the following section to provide a clear overview and serve as a foundation of the present paper. Currently, polymeric materials are used in a wide range of applications, from the automotive and naval industries to military and aerospace technology, biomedicine, construction, and the production of sporting equipment or consumer goods. Thus, polymers not only support technological and industrial development but have also become an integral part of culture and daily life [1,2,3,4]. One intensely studied aspect is the application of biodegradable polymers, given that process sustainability is an extremely important criterion; these polymers can be used in most of the applications discussed above [5,6,7].

Polymeric materials can be classified not only by their source, whether natural or synthetic, but also by their thermal behavior and stability. Depending on their behavior, a distinction is made between thermoplastic polymers, which can be reshaped by heating; thermoset polymers, which form three-dimensional networks and cannot be reshaped after cooling; and elastomers characterized by high elasticity and the ability to return to their original shape after deformation. This classification can also be supplemented based on applications, including fibers, plastics for molding, polymer composites, and adhesives. The concept of mechanical properties of polymeric materials refers to the material’s behavior under the action of external forces, such as tensile, compressive, flexural, or shear strength—properties that are essential for practical industrial applications [8,9,10,11].

Parts made of polymeric materials are produced through a variety of technological processes adapted to the material type and the part’s geometry. Among the most widely used are extrusion, injection molding, blow molding, thermoforming, pressing, and additive manufacturing (3D printing) [11,12,13]. Additive manufacturing (AM) constitutes one of the most common methods used to create structures and parts with complex geometries based on a three-dimensional digital model. The process unfolds through the successive deposition of material layers, which overlap to build the final shape of the part [14,15,16,17]. According to ISO and ASTM standards, this method involves building parts by adding material layer by layer, starting from 3D model data, thus differentiating itself from traditional techniques that involve either material removal or forming through pressing or molding [18].

Additive manufacturing encompasses a broad spectrum of technologies, with over 100 different processes reported in the literature [19]. According to several recent studies [20,21,22,23,24], these technologies are categorized into several main groups based on the method of material deposition or sintering. Thus, material extrusion (ME) includes techniques such as fused deposition modeling (FDM), also known as fused filament fabrication (FFF). Vat photopolymerization (VP) processes include technologies such as stereolithography (SLA), digital light processing (DLP), and projection stereolithography (PS). The powder bed fusion (PBF) category brings together processes such as electron beam melting (EBM), electron beam additive manufacturing (EBAM), selective laser sintering (SLS), selective heat sintering (SHS), direct metal laser sintering (DMLS), selective laser melting (SLM), and laser beam melting (LBM). Directed energy deposition (DED) includes processes such as laser metal deposition (LMD), direct metal deposition (DMD), direct laser deposition (DLD), and laser engineered net shaping (LENS). Other categories include binder jetting (BJ) and material jetting (MJ), with technologies such as multi-jet modeling (MJM), drop on demand (DoD), and thermo-jet printing (TJP), as well as laminated object manufacturing (LOM) and hybrid processes that combine additive methods with established techniques such as machining or welding [25].

The most widely used additive manufacturing process on a large scale is FDM, due to its technological simplicity, low costs, and application versatility [26,27,28]. This method does not require complex equipment or expensive materials, making it accessible to both the academic and research environments, as well as the industrial sector. FDM printers are easy to operate and maintain, and the availability of a wide range of materials—from PLA and ABS to technical polymers like PETG, PC, or fiber-reinforced composites—allows the process to be adapted to various applications [29].

An additional factor contributing to the popularity of FDM is its capacity for very fast prototyping and customization, offering an efficient balance between cost, speed, and precision. Furthermore, the development of FDM has been supported by an extensive community of users and open-source projects, contributing to the evolution of equipment, materials, and process-specific software. However, the method also presents certain limitations, such as high surface roughness, lower dimensional resolution compared to other additive manufacturing techniques (e.g., SLS or SLA), and anisotropic mechanical properties, which are lower in the direction of layer deposition. Additionally, the process can be relatively slow for large components, and the need for support structures involves extra time and material consumption [26,29,30].

The values of the mechanical properties of FDM parts can be modified by adjusting and optimizing the printing parameters, choosing the materials used, and applying post-processing methods. These three aspects influence the strength, hardness, elasticity, and durability of the parts. By combining them, the final mechanical behavior of the manufactured components can be controlled.

The mechanical performance of components made by FDM is critically determined by the stiffness of the interlayer bonds and the structural integrity of the deposited tracks. Recent research [31,32] demonstrates that reducing layer thickness and using negative spacing between tracks (overlap) are essential for minimizing internal porosity and increasing the tensile strength of the parts. In this context, thermal parameters play a fundamental role; maintaining appropriate temperatures at the nozzle and the build plate (60–90 °C) promotes molecular diffusion at the interface, improving cohesion and final mechanical performance [33]. At the same time, the intrinsic anisotropy of the process remains a major challenge, with building orientation and the raster angle (0°, 45°/−45°) being the determining factors for crack propagation patterns and impact resistance [34].

The evolution of FDM technology has led to the exploration of new strategies for enhancing stiffness and durability using composite materials or multi-materials. The integration of continuous fibers, such as glass or basalt, can significantly increase load-bearing capacity, while additives like metallic or organic particles enhance the elastic modulus, although there is a risk of reducing elongation at break [35,36]. Furthermore, hybrid approaches combining polymers with contrasting properties (e.g., PLA-TPU or ABS-TPU) allow for the creation of shape-memory structures and high fatigue resistance, provided there is precise control over interface adhesion and the layer deposition sequence [37]. Comparative analyses also indicate that printing speed and the ratio between nozzle diameter and layer thickness are critical variables directly influencing the balance between dimensional precision and flexural strength [38].

In addition to optimizing such parameters, the specialized literature highlights the importance of environmental conditions and post-processing techniques in defining the final mechanical profile. Printing in controlled environments, such as vacuum or nitrogen atmospheres, has proven effective in increasing tensile strength (by over 12%) by reducing oxidation defects [34]. Following fabrication, the application of thermal (annealing at 80–100 °C), chemical, or ultrasonic treatments can reduce surface roughness by up to 50% and eliminate residual voids, generating an increase in durability and tensile strength of up to 25% [2,33,36]. These post-processing interventions successfully compensate for the geometric limitations of the layer-by-layer deposition method, providing FDM parts with properties comparable to those obtained through traditional injection molding methods.

From the analysis of specialized works, it is evident that the mechanical properties of FDM parts significantly depend on the process operating conditions. The general aspects highlighted include the influence of layer thickness, printing speed and temperature, infill density, part orientation, and raster angle on tensile strength, elastic modulus, and interlayer cohesion.

Aspects less highlighted to date refer to the complex interaction between multiple parameters simultaneously, such as the combined influence of layer orientation, speed, and temperature on material anisotropy and crack propagation, as well as how post-processing modifies the effects of initial printing parameters on the final characteristics of the parts. Additionally, research on real parts in practical applications remains limited compared to studies on standardized specimens, which leaves many unknowns regarding the actual mechanical behavior of components manufactured through FDM.

This paper aims to provide a comprehensive systemic analysis of how FDM operating conditions influence the mechanical properties of homogeneous thermoplastic polymers (such as PLA, ABS, TPU, and PEEK). The primary contribution lies in establishing a critical ranking of these influencing factors and exhaustively integrating experimental results—encompassing tensile, compressive, flexural, torsional, impact, and fatigue strength, as well as hardness and vibration-damping capacity—with advanced numerical simulation (FEM/FEA) and mathematical modeling techniques for property optimization. To ensure scientific rigor, a structured methodological framework was applied for literature selection, querying major academic databases (such are Web of Science, Scopus, SpringerLink) for peer-reviewed articles that provide quantitative validation of these interdependencies. The selected literature predominantly features recent works (under 5 years) to reflect the current state of the art, complemented by foundational studies highlighting the field’s technological evolution. Reflecting this scope, the work logically progresses from a fundamental presentation of polymeric materials and the FDM process, through the systemic analysis and ranking of input parameters, to the detailed synthesis of mechanical properties, numerical/mathematical modeling, and future evolutionary trends.

## 2. Polymeric Materials and Their Mechanical Properties

In FDM technology, polymers are used in the form of thermoplastic filaments that are melted and extruded layer by layer to obtain three-dimensional parts, with their performance being strongly influenced by processing parameters and the presence of internal defects [36]. In the specialized literature, these polymers are described as having limitations in mechanical properties compared to conventional manufacturing methods, which is why material selection and understanding of their internal structure is essential [36,39]. The classification of polymeric materials used in FDM 3D printing can be made primarily according to their network structure and degree of crystallinity, these structural characteristics being directly correlated with the mechanical response of the resulting parts [40,41].

From the perspective of network structure, linear polymers are characterized by continuous chains without branches, with PLA being a representative example. PLA is a biodegradable biopolymer obtained from renewable resources, widely used in FDM due to its good processability, dimensional stability, and high interlayer adhesion [42]. Branched polymers feature main chains from which side branches extend, such as low-density polyethylene (LDPE), highlighted in experimental studies as a material with high flexibility and deformability, suitable for applications requiring ductile behavior [43]. Crosslinked polymers form three-dimensional networks through covalent bonds between chains, with epoxy materials used as polymer matrices in FDM applications often being subjected to post-curing treatments to increase stiffness and mechanical stability [36]. Cellular polymers, such as certain polyurethane (PU) foam systems, are characterized by a porous structure, which allows the production of lightweight components with good insulation and vibration-damping properties [44].

Regarding the state of crystallinity, crystalline or semi-crystalline polymers exhibit ordered regions at the molecular level, an aspect that gives them a well-defined melting point and specific mechanical behavior; glycol-modified polyethylene terephthalate (PETG) is reported in the specialized literature as a material with good ductility, transparency, and dimensional stability in the FDM process [44], while PA12 (Nylon 12) is known for its abrasion resistance and favorable behavior in functional applications [45]. Amorphous polymers are characterized by a disordered structure and the presence of a glass transition, relevant examples in the FDM context being acrylonitrile butadiene styrene (ABS), valued for its impact resistance, polycarbonate (PC), known for its high toughness and transparency, and ASA, frequently used for applications exposed to outdoor environments due to its stability against UV radiation [46]. In the category of high-performance polymers is PEEK, a semi-crystalline thermoplastic with high mechanical and thermal resistance, which, when printed via FDM under suitable temperature conditions and in a controlled environment, allows the production of components intended for demanding applications in fields such as medicine or aerospace [39,47].

For the classic polymers used in FDM technology, the relevant mechanical properties are primarily tensile strength, compressive strength, flexural strength, torsional strength, impact strength, fatigue strength, and hardness. These describe the material’s response to different types of loading and allow for the evaluation of the behavior of additively manufactured parts [48,49]. In the FDM context, these properties are influenced by the anisotropy induced by layer-by-layer deposition, printing parameters, and testing conditions [41,50]. Tensile strength is defined as the maximum stress the material can withstand under uniaxial tension before failure, typically measured using the ASTM D638 test [51], and is used to characterize the stiffness, ductility, and maximum allowable load of FDM parts [49]. Compressive strength indicates the ability to withstand axial forces without excessive permanent deformation, being determined according to ASTM D695 [48,52]. Flexural strength measures stiffness under transverse load through three-point or four-point bending tests, according to ASTM D790 [53], and is essential for beams, plates, and components subjected to bending [50]. Torsional strength represents the maximum torque sustained under twisting without failure, referenced in ASTM D1043 [54], and is necessary for shafts, axles, or tubes printed via FDM [48]. Impact strength quantifies the energy absorbed under sudden impact (Charpy or Izod tests, ASTM D256 [55]), defining the toughness and ability of parts to withstand shocks in dynamic applications [56]. Fatigue strength describes the number of cycles of repeated loading until failure, evaluated according to ASTM E466 [57], and is essential for components subjected to vibrations or cyclic loads [48]. Finally, hardness measures resistance to localized indentation, being determined using Shore D scales, ASTM D2240 [58], or Rockwell, and is used to assess the wear and abrasion resistance of the surfaces of FDM parts [48,49].

The mechanical properties of polymers differ significantly from those of metals, both in absolute values and in sensitivity to the environment and the manufacturing process. In general, polymers have a much lower density, elastic modulus and tensile strength but can exhibit higher elongation at break and better vibration damping, which makes them desirable for lightweight components and applications where mass reduction is essential [48,49]. Metals, such as steel or aluminum alloys, exhibit high values of Young’s modulus and yield strength, with largely isotropic behavior, so that stiffness and load-bearing capacity are significantly superior to those of polymers, especially at high temperatures [41,49]. However, the comparison of specific properties, normalized by density, shows that certain high-performance polymers, such as PEEK, can achieve strength-to-weight ratios close to or even exceeding those of some light metal alloys [48].

## 3. Modifying the Mechanical Properties of Materials Manufactured Through the FDM Process

For FDM technology, manufacturing begins with a three-dimensional CAD model that is sliced into successive layers using slicing software, resulting in a file containing the deposition trajectories of the thermoplastic material [14]. The thermoplastic filament is continuously fed into the print head, where it is brought to a molten or semi-molten state and then extruded through the nozzle, being deposited layer by layer onto the build platform according to the digitally defined geometry [48]. During deposition, the molten material cools and partially solidifies, fusing with the previous layer, which determines interlayer adhesion and, implicitly, the final mechanical properties of the part [14,49].

**Figure 1:**
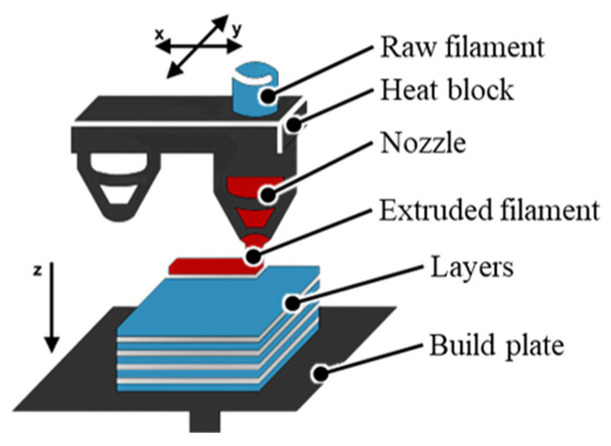
FDM part building principle (adapted after [59]).

Figure 1 schematically illustrates the principle of the FDM process. The filament is introduced into the extrusion head, heated, then extruded through the nozzle and deposited in the form of successive layers onto the build platform, with relative movement along the *x*–*y*–*z* axes allowing for the attainment of the desired three-dimensional geometry [59]. Typically, the FDM printer includes motion mechanisms for positioning the nozzle, a temperature control system for the nozzle and bed, as well as possible support materials that are deposited simultaneously with the base material to support overhangs [48]. FDM can be viewed as a numerically controlled capillary extrusion process, in which part quality is the result of the interaction between the digital model, the deposition path, and the local thermal conditions [14].

In the specialized literature, the technological workflow of the FDM process is generally divided into three main stages: pre-processing (model preparation and print settings), production (the actual printing), and basic post-processing (handling and cleaning of parts) [14,60]. In the pre-processing stage, the process starts with the three-dimensional CAD model, which is exported into a format compatible with the printer, typically STL or OBJ, and imported into slicing software [14]. Within the slicer, the part orientation on the platform, layer thickness, infill density, path type (infill and contour), and the need for support structures, as well as the material and nozzle diameter, are established. Subsequently, the G-code file containing the movement and extrusion instructions is generated [14]. In parallel, equipment preparation operations are carried out, such as calibrating and leveling the print bed, loading the filament, and verifying the operation of the nozzle and heating systems [60]. The production stage initially comprises the heating phase of the nozzle and bed to the set temperatures, followed by the layer-by-layer printing phase according to the generated code, where the printer successively deposits the base material and possibly the support material until the part is completed [14,61]. Throughout this process, monitoring operations and minor interventions may be necessary, for example, stopping the print in case of delamination, filament shortage, or part detachment from the bed [49]. Finally, in basic post-processing, the part is detached from the bed, support structures are removed mechanically or, as applicable, dissolved, and the surfaces are cleaned of any burrs or material residues before applying more advanced additional processing, described in the following sections [14,49].

FDM parts already exhibit anisotropic mechanical behavior due to microporosity and imperfect bonding between layers, and post-processing interventions act upon this heterogeneous microstructure [61].

Annealing treatments applied to FDM parts, for example, made of ABS, can reduce residual stresses and promote microstructural rearrangements (reduction of microvoids, increase in the degree of crystallinity), leading to better interlayer bonding and increased tensile strength and interlaminar toughness [62,63]. In the study by Lluch-Cerezo et al. for ABS, it was shown that, under more severe annealing conditions, interlaminar toughness can increase significantly (by over an order of magnitude), precisely through the closure of internal voids and the elimination of residual stresses [62]. However, the same study shows that annealing produces significant dimensional deformations (shrinkage in length, variations in width and height), which can render parts unusable if treated “in air,” without support [62]. Introducing a ceramic powder mold around the parts drastically reduces deformations (effectiveness over 80–90% depending on geometry) but does not eliminate the risk of distortions and requires careful control of temperature and treatment time [62,63].

Diniță et al. present numerous studies in which chemical treatments (for example, solvent vapor smoothing for ABS) strongly reduce surface roughness and mitigate the “staircase” effect of the layers, which can diminish the role of surface stress concentrators and improve fatigue behavior [63]. In some cases, moderate increases in static strength (higher tensile strength at break) are also reported after solvent smoothing, if the parameters are well controlled (temperature, time, uniform exposure). However, the same review emphasizes that chemical treatments can also lead to a decrease in strength if the surface layer is excessively plasticized, microcavities appear, or material mass is lost, generating a surface zone weaker than the core. Non-uniform treatments (for example, only on certain easily accessible faces) create a gradient of properties across the thickness and surface of the part, introducing a new form of anisotropy and potential stress concentrators in the transition zones [63].

Sawant et al. describe in detail the effects of sanding, blasting, and vibratory finishing on FDM parts. These processes reduce roughness, eliminate sharp edges and obvious layer lines, and, in principle, can improve fatigue strength by reducing surface stress concentrators [64]. The authors show, however, that manual sanding is difficult to control repeatably, and excessive material removal, especially in the outer perimeters, which carry a large portion of the load, can lead to the appearance of microcracks and a decrease in overall strength. The study by Wikło et al. on PET-G components shows that mechanical properties (longitudinal modulus, tensile strength) are sensitive to the internal structure and the quality of interlayer bonds; aggressive mechanical post-processing, which “exposes” internal porosity or breaks material fibers at the surface, can make these defects mechanically active and significantly reduce the load at failure [65].

Bol and Šavija show that FDM parts already exhibit pronounced anisotropy, determined by layer orientation, deposition parameters, and non-uniform internal porosity [61]. Post-processing (thermal, chemical, mechanical) modifies this microstructure: some well-controlled treatments can reduce anisotropy (for example, by improving interlayer bonding), but non-uniform or overly aggressive treatments can create new local imbalances [61,63]. One example is annealing: it can increase interlayer adhesion and reduce differences in properties between directions but at the same time can generate large deformations and redistributed residual stress fields, especially in thin geometries or those with abrupt cross-sectional changes [62]. Similarly, solvent treatments can homogenize and strengthen the surface layer but can create a “skin” layer with properties different from the core, which complicates the response to complex loads [63,66].

Diniță et al. emphasize that post-processing can substantially improve the mechanical properties (tensile strength at break, toughness, fatigue behavior) and dimensional stability of additively manufactured components, but only if chosen and controlled in correlation with the material, geometry, and application [63]. The explicit recommendation is that, for structural applications, treatments should be experimentally validated on representative test specimens and applied uniformly, so that the benefits (better interlayer bonding, reduced porosity, lower roughness) are not counterbalanced by excessive deformations, material loss, or zones with properties very different from the rest of the part [62,64].

The modification of mechanical properties in FDM parts is the direct result of how process parameters control interlayer bonding, porosity level, and the anisotropic architecture of the part [14,33]. Key parameters—nozzle and bed temperature, printing speed, layer height, orientation and raster angle, infill density and type, as well as air gap and wall thickness—act together on these mechanisms and, consequently, on strength, stiffness, and toughness [14,67].

The study by Abouzaid et al. shows that extrusion temperature is one of the dominant factors for porosity and mechanical properties: too low temperatures lead to weak interlayer bonding, delamination, and large interlayer pores, while moderate to high temperatures increase the time spent above the glass transition temperature and improve interlayer fusion [67]. Results synthesized by Gao et al. confirm that extrusion temperature and platform temperature directly influence the thermal field within the part, interlayer and intralayer cohesion, and consequently, tensile, compressive, and flexural strength [33]. At excessively high temperatures, local thermal degradation can occur, viscosity may decrease to the point of difficult-to-control flow, and stringing and dimensional distortions can appear, which generate imprecise geometry and potential stress concentrations [67]. Conversely, an optimal temperature regime reduces effective porosity (smaller and fewer voids at the layer intersections), increases the actual contact area between filaments, and reduces the contrast between properties along and perpendicular to the layering direction, i.e., it mitigates mechanical anisotropy [61,67].

Printing speed controls the time during which the extruded material remains hot and capable of adhering to the previous layer; high speeds reduce this time, which weakens interlayer bonding and increases effective porosity [33]. Gao et al. explicitly mention that increasing printing speed, together with increasing layer height, leads to diminished ductility and, in some cases, a decrease in strength, especially for vertical orientations where the load is nearly perpendicular to the layers [33]. At moderate speeds, heat transfer between layers is more efficient, which favors polymer chain diffusion and a larger bonding area, thus resulting in greater interlayer strength [67]. However, excessively low speeds can lead to local overheating, thermal accumulation, and deformations (edge curling, “elephant foot”), so the relationship between speed and mechanical properties is non-linear and must be optimized together with temperature [14,33].

Layer height influences both the number of layer-to-layer interfaces as well as the geometry of the material bead and the overlap area between layers [67]. Abouzaid et al. show that thin layers (below approximately 0.3–0.4 mm for the polymers studied) generally lead to better mechanical properties, as they reduce pore size and facilitate a more uniform stress distribution, even though the number of interfaces is greater [67]. Gao et al. synthesize several studies in which increasing layer height reduces ductility and, for certain orientations, also decreases strength [33]. The “air gap” parameter (the distance between parallel paths) is closely related to porosity: a positive air gap produces voids between layers and low density, a zero air gap minimizes porosity, while a negative air gap leads to layer overlap and high density but can affect surface quality [67].

Build orientation and layer angle are recognized as the most important factors for how mechanical anisotropy manifests in the part [33,67]. Abouzaid et al. show that flat orientation (construction in the XY plane) generally maximizes Young’s modulus and tensile strength, while vertical orientations (load nearly perpendicular to the layers) highlight the weakness of interlayer bonds and lead to much lower values of strength and toughness [67]. The micromechanical models discussed by Bol and Šavija show that interfaces between layers and layer orientation determine the principal stiffness directions and preferential fracture planes; angles such as 0°/90° or ±45° lead to different crack propagation paths and different toughness values [61]. From an experimental perspective, results compiled by Gao et al. and Ahmad et al. show that orientation and deposition angle of layers have a stronger impact on tensile and flexural strength than other parameters, such as speed or infill pattern, especially at high infill densities [14,33].

Infill density directly controls the solid/void volume fraction in the part: low density reduces mass and printing time but increases overall porosity and reduces the effective load-bearing cross-section, leading to a decrease in strength and stiffness [33]. Gao et al. emphasize that, at high densities (nearly solid), the influence of other parameters (infill pattern, speed) becomes relatively smaller, while at low densities, variations in the infill pattern (e.g., hexagonal vs. rectilinear) can change the fracture mode and strength values [33]. Abouzaid et al. highlight that effective density is the primary parameter for obtaining functional parts: high values, combined with zero or negative air gap, reduce porosity, increase the contact area between filaments, and significantly increase tensile strength and elastic modulus [67]. Wall/perimeter thickness plays a complementary role: in specimens with the same infill, a greater number of outer perimeters increases the contribution to load bearing, which was observed, for example, in tensile tests on PET-G specimens by Wikło et al., where the perimeter structure clearly influences the tensile response [65].

From a conceptual perspective, Gao et al. classify FDM parameters into three groups: process parameters (temperature, layer thickness, speed, infill density, etc.), environmental parameters (bed temperature, chamber temperature), and other printer parameters (nozzle diameter, etc.), showing that they act together on inter- and intralayer adhesion and on the formation of porosity [33]. Ahmad et al. synthesize dozens of studies and show that parameters such as orientation, layer deposition angle, infill density, layer height, and extrusion temperature are the most frequently reported as having significant effects on tensile, compressive, flexural, and impact strength [14]. Abouzaid et al. and Bol and Šavija converge on the idea that anisotropy, porosity, and interlayer adhesion must be considered together: modifying temperature, layer height, or air gap changes the pore structure and the thickness of the bonding zones, which affects both the average values of mechanical properties and the preferential fracture directions [61,67]. In practice, optimizing the FDM process for mechanical properties, therefore, involves finding a compromise between: reducing porosity (appropriate temperatures, dense infill, small air gap), controlling anisotropy (orientation and raster angle suitable for the load directions), and limiting geometric distortions or defects associated with extreme parameters [14,33].

It is also worth noting the existence of some works in which contradictory results are found regarding the influence of some input factors in the FDM process on the characteristic values of some mechanical properties of polymeric materials. Thus, one can find research in which it is found that increasing the thickness of the deposited layer contributes to an increase in the resistance to mechanical stress, while in other research, it is estimated that the effect is negligible or even manifests itself in the opposite direction [32]. Pronounced differences of opinion can be observed regarding the factor with the most significant influence on the mechanical properties of polymeric materials, some researchers assessing that this could be the infill density or the thickness of the layer, while other researchers consider that the orientation of the printing directions would be the dominant factor or indicate the existence of some maxima or minima [34,38]. Situations are also mentioned in which excessive pursuit of improving one property may result in the negative impact of another mechanical property [38].

## 4. Systemic Analysis of FDM Parameters’ Effect on Mechanical Properties

The concept of systemic analysis refers to approaching a process as a system composed of interconnected elements, in which the relationships between input factors, intermediate processes, and outcomes are studied, along with their interactions. It does not analyze an isolated parameter, but rather the entire ensemble of influences and cause-and-effect connections.

**Figure 2:**
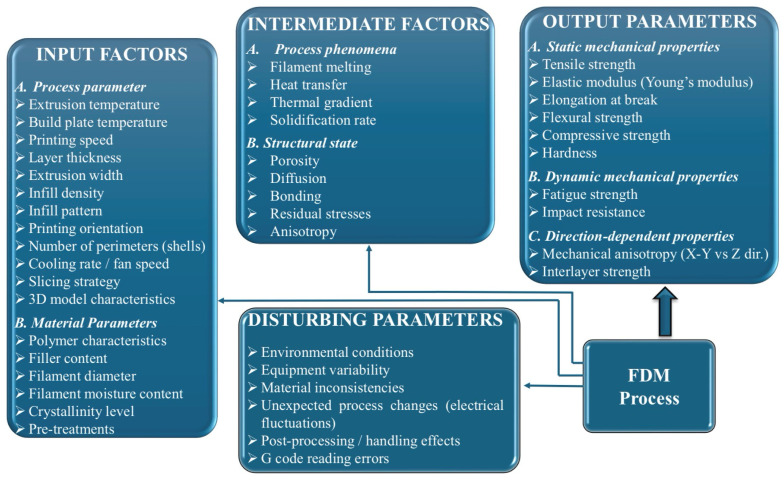
Systemic analysis of the FDM process: interdependencies between input parameters, intermediate phenomena, and final mechanical properties.

Thus, output parameters represent the dependent variables that define the structural integrity and functional performance of components manufactured through 3D printing, being a result of the complex interactions between deposition kinetics and heat transfer phenomena. These are classified according to the material’s response to external loads, starting with static mechanical properties, such as tensile strength, modulus of elasticity (Young’s modulus), and elongation at break, which quantify stiffness and load-bearing capacity. Of critical importance in an industrial context are dynamic properties, especially impact and fatigue strength, which determine the durability of the part under variable operating conditions [20,21,38]. Systemic analysis (Figure 2) highlights, however, that these indicators are governed by mechanical anisotropy and interlayer strength, parameters that reflect the quality of polymer fusion at the interface of successive depositions; the latter are defining for characterizing the part’s behavior in different directions, being indicators of the success of optimizing FDM process parameters.

The input factors in the FDM process are classified into two main groups: process parameters and material parameters. The first category includes software-controllable variables, such as extrusion temperature, deposition speed, layer thickness, infill density, part orientation on the platform, etc. The group of material parameters includes filament properties, such as polymer composition, filament diameter, and moisture content, all of these factors determining the final part performance. The influence of these input factors on mechanical properties is mediated by intermediate factors, which represent the physical phenomena occurring during part fabrication. Finally, the interaction between these parameters defines the output values, particularly tensile strength and impact strength. At the same time, the mechanical anisotropy of the material is dictated by the printing orientation, because the bonds formed through fusion between layers are, in general, the critical point where structural failures occur under mechanical loading [9,34,68,69,70].

## 5. Ranking of Influencing Factors of Mechanical Properties of Polymer Materials When Using the FDM Process

FDM process parameters that influence mechanical properties can be systematically categorized into six primary groups: material characteristics, process conditions, slicing parameters, printing equipment, operator decisions, and 3D model quality [34,71,72]. The literature review across multiple studies highlights print orientation, layer height, infill density, raster angle, and extrusion temperature as the most critical parameters affecting mechanical performance [34,73,74].

Print orientation emerges as the single most critical parameter, producing a 1.5–3× strength differential between optimal and suboptimal orientations [73]. This effect reflects the fundamental anisotropy inherent to FDM parts: components printed with loading parallel to layers (XY orientation) show superior strength, while those loaded perpendicular to layers (Z orientation) fail in weaker loading scenarios. Even within the same material (ABS), orientation selection produces strength variations from 22.51 MPa (Y-axis) to 35.45 MPa (X-axis), representing a 57% performance swing [73]. Kopar et al. [71] confirmed, through coupled numerical–experimental analysis, that printing direction governs residual stress distribution and deflection, with the 45°/−45° raster orientation yielding the highest tensile performance in ABS FFF specimens compared to 0°/90° and other configurations.

**Figure 3:**
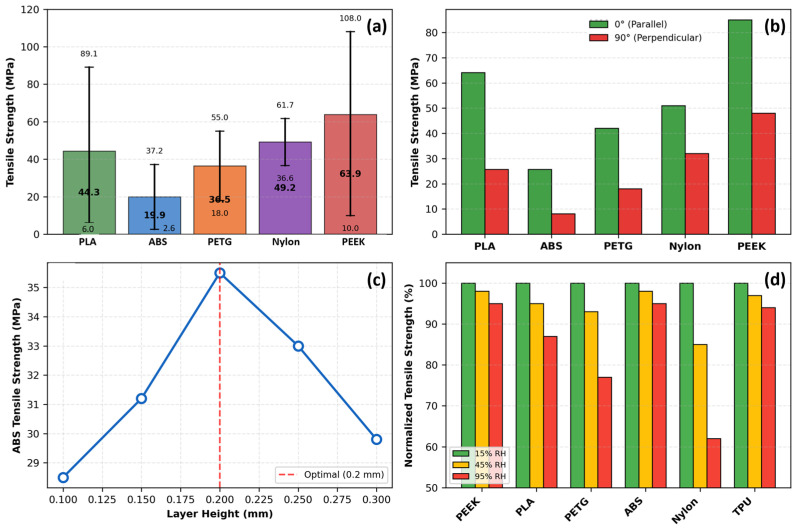
Analysis of tensile strength in FDM 3D-printed thermoplastic polymers. (**a**) Range and average tensile strength by material with error bars indicating full min–max range. (**b**) Raster angle effect comparing parallel (0°) and perpendicular (90°) orientations across five materials, demonstrating 60–68% strength reduction at 90°. (**c**) Layer height influence on ABS tensile strength showing optimal performance at 0.2 mm. (**d**) Relative humidity effects on six materials at 15%, 45%, and 95% RH expressed as percentage of 15% RH baseline strength. Data synthesized from: [71,72,73,76].

Layer height follows as the second most influential parameter, though its effect is more nuanced than simple linear relationships indicate. For ABS, optimal performance occurs at 0.2 mm layer height, with both smaller and larger values yielding reduced strength, as illustrated in Figure 3c. Reducing layer height from 0.3 mm to 0.1 mm increases PLA tensile strength by 15–25% through increased interlayer bonding area and reduced stress concentrations at interfaces [34]. Conversely, this benefit diminishes at low infill densities where failure becomes dominated by the infill structure rather than interlayer bonding, highlighting the critical importance of parameter interactions that single-factor studies systematically overlook [73]. Hsueh et al. [72] fixed layer height at 0.2 mm for both PLA and PETG across all temperature–speed combinations, reporting that this mid-range setting delivered the most consistent mechanical outcomes across multiple loading modes (tensile, compression, and bending).

Infill density demonstrates a strong but non-linear correlation with mechanical properties, with diminishing returns above 70–80% density. Increasing infill from 20% to 100% raises tensile strength by 50–150%, depending on material and pattern selection [73]. Hozdić and Hasanagić [75] quantified this relationship precisely for PLA: tensile strength increased from 22.49 MPa at 40% infill to 45.00 MPa at 100% infill (a 100.09% enhancement), while Young’s modulus increased from 595.2 MPa to 1051.2 MPa over the same range. Critically, this relationship plateaus because tensile failure in FDM parts typically initiates at interlayer interfaces rather than within the infill structure itself [73,75], confirming that simply adding more material cannot overcome the intrinsic weakness of interlayer bonding.

Raster angle influences mechanical anisotropy, as visualized in Figure 3b. For PLA, parallel orientation (0°) achieves 64.1 MPa, while perpendicular orientation (90°) reaches only 25.7 MPa—a 60% strength reduction. ABS exhibits even greater sensitivity: 25.7 MPa at 0° versus 8.1 MPa at 90°, a 68% reduction [73]. Ramírez-Prieto et al. [77] confirmed these trends for PLA+ filament, demonstrating that parallel raster (0°/0°) produced the highest tensile strength, while the crisscross orientation (45°/−45°) yielded the lowest tensile strength but the greatest ductility—attributable to favorable shear deformation dynamics along the raster plane. Alternating ±45° raster angles provide a practical compromise, delivering 70–80% of maximum strength while offering more isotropic behavior [34].

Extrusion temperature directly controls polymer melt viscosity and thermal diffusion at interlayer interfaces. Higher temperatures within the recommended range consistently improve interlayer bonding. Gao et al. (2015) [73] reported a 20% strength increase for PLA when the temperature rose from 190 °C to 220 °C. Hsueh et al. [72] demonstrated that PLA and PETG mechanical properties (tensile, compressive, and bending) increase monotonically with printing temperature across the 180–220 °C (PLA) and 225–245 °C (PETG) ranges, with the relationship attributed to decreased melt viscosity and improved interlayer fusion at elevated temperatures. Excessive temperatures, however, risk thermal degradation, stringing, and dimensional inaccuracy, necessitating material-specific optimization [78].

The tensile strength across thermoplastic materials reveals extraordinary potential alongside consistent variability, as highlighted in Figure 3a. PEEK dominates the strength spectrum with an average of 63.9 MPa and a maximum of 108.0 MPa, though its 98.0 MPa range reflects both a peak performance level and extreme sensitivity to processing conditions [73]. PLA, the workhorse of desktop 3D printing, exhibits an average tensile strength of 44.3 MPa with maxima reaching 89.1 MPa; however, PLA’s tensile strength spans an 83.1 MPa range (6.0–89.1 MPa), such that the weakest specimen possesses only 7% of the strength of the strongest [73]. Hozdić and Hasanagić [75] corroborated this sensitivity in a controlled study, reporting PLA tensile strengths from 22.49 MPa to 45.00 MPa across infill densities of 40–100%, with Young’s modulus spanning 595–1051 MPa, a variability driven exclusively by a single parameter.

**Figure 4:**
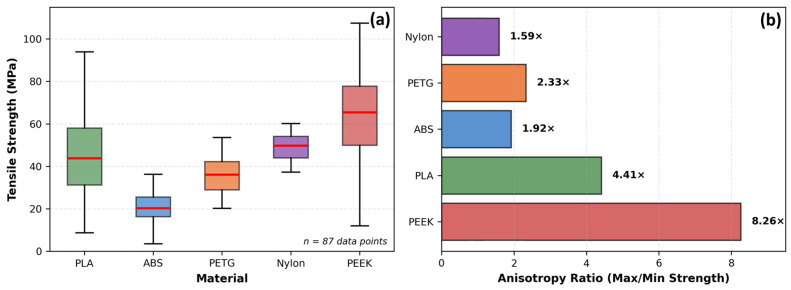
Analysis of tensile strength distributions and mechanical anisotropy. (**a**) Box plots showing median, interquartile range, and full range for five materials compiled from multiple open-access experimental studies, illustrating that PLA specimens range from 6.0 to 89.1 MPa. (**b**) Mechanical anisotropy ratios (maximum/minimum strength across orientations) demonstrating PEEK’s extreme directional dependence (8.26×), PLA’s moderate anisotropy (4.41×), and ABS’s relatively low anisotropy (1.92×). Data compiled from: [72,73,75,79].

Mechanical anisotropy ratios vary dramatically by material type, as shown in Figure 4b: PEEK exhibits an 8.26× anisotropy ratio, PLA a moderate 4.41×, and ABS a relatively low 1.92× [73]. Recent work on PEEK by Liu et al. [79] demonstrated that printing path exerts a dominant influence on anisotropic behavior, with elongation at break varying by up to twofold across build orientations (reaching a maximum of 96%), while specimens printed along the W or T paths exhibited elongations at break below 5%. This pattern confirms that high-performance polymers, despite their superior absolute strength, exhibit increased sensitivity to processing decisions.

Environmental factors, such as moisture, exert effects comparable in magnitude to processing parameters, yet remain systematically underexplored. As shown in Figure 3d, Sun et al. [76] examined six FFF polymers acclimatized at 15%, 45%, and 95% relative humidity, demonstrating that Nylon exhibits a 30–40% tensile strength reduction from the lowest to the highest humidity level, PETG degrades by 20–25%, and PLA suffers 10–15% losses. Hamrol et al. [80] specifically investigated ABS filament, finding that even ambient moisture levels (0.1–0.3% by weight) reduce tensile strength by 5–10% and increase porosity, recommending drying at 80 °C for 4–6 h as a minimum quality assurance step. The mechanism involves hydrolysis during printing and steam bubble formation that disrupts interlayer adhesion, creating internal defects invisible to external inspection [76].

Polymer grade variability compounds environmental challenges. Alhuzaim [81] tested PLA filaments under varying processing conditions, demonstrating that tensile strength spans 35–45 MPa under otherwise identical conditions. The variability in FDM conditions is due exclusively to differences in molecular weight, additives, and processing history among filament batches. Hozdić and Hasanagić [75] noted that PLA and PLA+CF specimens from the same manufacturer delivered manufacturer-specified tensile strengths of 45–49 MPa and 40–45 MPa (ISO 527) [82], respectively, with experimental values at 100% infill (45.00 and 42.54 MPa) aligning closely with these specifications—underscoring the importance of material source documentation in any reproducible study.

Parameter interactions represent the most underexplored dimension of FDM optimization. The benefit of reducing layer height from 0.3 mm to 0.1 mm proves highly dependent on infill density: at high infill (>70%), the reduction yields substantial strength improvements, yet at low infill (<40%), the effect diminishes to less than 5% [73]. Similarly, each 20 mm/s increase in printing speed requires approximately 5–10 °C temperature elevation to maintain equivalent interlayer bonding quality [73]. Kopar et al. [71] demonstrated that residual stresses at the interface between the build platform and the first deposited layers are substantially higher than in upper layers; a finding with direct implications for the observed dependence of mechanical properties on both temperature and speed settings.

**Figure 5:**
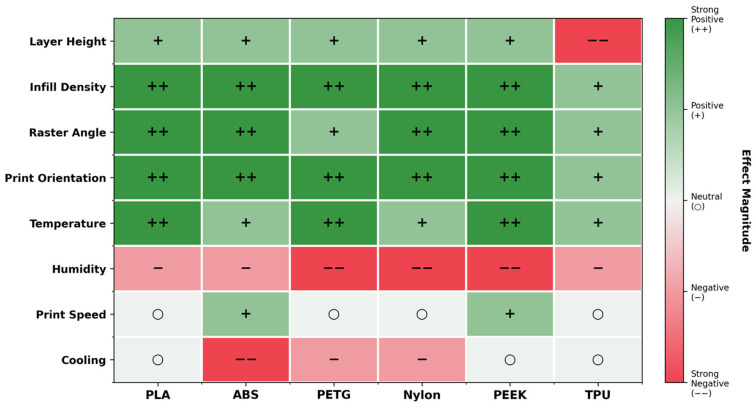
Heatmap of processing parameter effects on tensile strength for six pure thermoplastic materials. Color scale: strong positive (++, dark green), positive (+, light green), neutral (○, gray), negative (−, light red), strong negative (−−, dark red). Parameters evaluated: layer height, infill density, raster angle, print orientation, extrusion temperature, relative humidity, print speed, and cooling intensity. Data synthesized from: [34,72,73,76,79].

Figure 5 provides a comprehensive heatmap synthesizing parameter effects across six materials, revealing material-specific response patterns that defy universal optimization rules. PLA exhibits strong positive responses to infill density and temperature (denoted ++) yet shows neutral behavior for cooling; a stark contrast to ABS, which requires minimal cooling (strong negative effect, −−) to prevent warping. PETG demonstrates strong negative humidity sensitivity (−−) while maintaining neutral print speed response, indicating that environmental control proves more critical than production speed optimization for this material. TPU’s strong negative response to layer height (−−) indicates that the typical strategy of reducing layer height for strength improvement proves counterproductive for elastomeric materials [34,73].

The interaction between raster angle and print orientation produces multiplicative effects: combined optimization can achieve 2–3× strength improvements versus suboptimal combinations, exceeding the sum of individual parameter effects [34]. Furthermore, higher extrusion temperatures can partially compensate for moisture-induced strength losses in hygroscopic polymers, suggesting that real-time parameter adjustment based on environmental sensing could mitigate quality variations [76].

## 6. Evolution of Research on the Influence of FDM Process Conditions on the Mechanical Properties of Polymer Materials

An analysis of how researchers have approached the issue of the influence exerted by input factors in the FDM process on the mechanical properties of polymeric materials highlights the existence of several stages, the most important of these stages being those mentioned below.

1. *The stage of exploratory studies* (approx. 2005–2012), in which the influence of a single input factor was considered, primarily on the tensile strength of a material, which, in frequent cases, was polylactic acid and acrylonitrile butadiene styrene. The input factors were printing orientation, layer thickness, and infill density. The pronounced anisotropy of the material and the important influence exerted by the printing direction were highlighted [83].
2. *The stage of conducting multifactorial studies and initiating the first attempts to optimize the FDM process* (approx. 2012–2018). The design of experiments method, the Taguchi method, and the response surface method were used. Factors such as nozzle temperature, bed temperature, printing speed, and infill pattern were considered. Among the mechanical properties analyzed were tensile strength, modulus of elasticity, flexural strength, and impact strength. Important interactions between input factors in the FDM process were highlighted. Research aimed at optimizing manufactured parts by maximizing their strength or stiffness was undertaken [84,85,86].
3. *The stage of approaching the microstructure and numerical modeling level* (approx. 2016–2021). Correlations between the material microstructure in parts manufactured via the FDM process and the mechanical properties of the material were studied. Attention was given to material porosity, crack initiation mechanisms, and models that considered material anisotropy were developed [87,88].
4. *The stage of approaching advanced materials* (approx. 2015–present). The mechanical properties of composite materials with reinforcing elements made of short fibers (carbon, glass), long fibers, or high-performance materials such as polyetherimides (ULTEM) or polyether ether ketone (PEEK) were studied. The focus was primarily on improving adhesion between deposited layers, controlling properties along specific directions, predictive modeling using machine learning, and multi-criteria optimization considering, for example, mechanical strength, mass, and process duration [14,38,89,90,91,92,93,94,95].
5. *The stage of an integrated approach and an expansion of industrial applications* (approx. 2020–present). Standardization of testing methods occurred [49]. Correlations between process, structure, properties, and performance in use were established [96]. Research results were validated through applications that considered material structure. Research aimed to obtain information regarding process repeatability, environmental influence (humidity and temperature), material fatigue strength, and part reliability.

A synthesis of results considered important from research aimed at highlighting the influence of FDM process conditions on the mechanical properties of polymeric materials is presented in Table 1.

This section also synthesizes, through graphical representations, the main evolution trends in research dedicated to the FDM process and the mechanical properties of polymeric materials. Below are two graphical representations highlighting the number of scientific articles identified on this topic from the year 2000 to the present, following the analysis conducted based on the Google Scholar and ScienceDirect databases.

Given that PLA is one of the most widely used materials in the FDM process, a synthesis was carried out of the number of scientific papers from the last 10 years in which its mechanical properties were analyzed.

**Figure 6:**
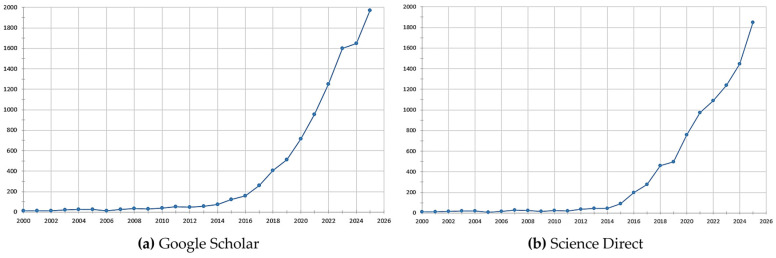
The number of articles indexed in Google Scholar (**a**) and in ScienceDirect (**b**) for the FDM process and the mechanical properties of polymeric materials.

Figure 6, which synthesizes the number of scientific articles indexed in Google Scholar and, respectively, ScienceDirect, on the general topic of the FDM process and the mechanical properties of polymeric materials, reveals an exponential increase in scientific interest over the last two decades. Between 1867 and 2000, only 10 articles were recorded in each of the two databases. By 2025, the number of recorded works had reached almost 2000. The growth becomes visible starting in 2015 and intensifies strongly after 2020, reflecting both the popularization of FDM technology at an industrial and academic level as well as the maturation of the additive manufacturing field. Although Google Scholar records slightly higher values due to the inclusion of diverse sources such as conference papers, preprints, or theses, both platforms indicate the same upward trend, confirming the consolidation of research in high-impact publications.

**Figure 7:**
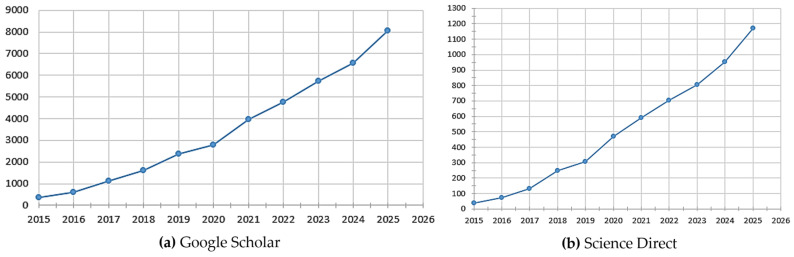
The number of articles indexed in Google Scholar (**a**) and in ScienceDirect (**b**) for the FDM process and the mechanical properties of PLA in the last 10 years.

A specific research direction that has experienced remarkable development in the last ten years is the analysis of the mechanical properties of PLA within the FDM process. Thus, following the synthesis carried out based on Figure 7a,b, it can be observed that the number of articles dedicated exclusively to this subject has grown spectacularly starting in 2015. If in 2015 approximately 400 works were registered in the Google Scholar database and 40 in the ScienceDirect database, by 2025, there were 8100 works registered in the Google Scholar database and, respectively, 1160 works registered in the ScienceDirect database. The most pronounced acceleration occurs after 2019, indicating an increasing focus of the scientific community on optimizing process parameters for PLA, a material that is among the most used in FDM technology due to its accessibility and versatility. The quantitative difference between the two databases is again explained by the nature of the sources included, but the common trend of sustained growth confirms that the study of the mechanical properties of PLA in FDM represents a consolidated and expanding research direction.

Overall, the comparative analysis of the four graphical representations provides a synthetic picture of the evolution of research in the field, highlighting both the growing interest in FDM technology and the mechanical properties of polymeric materials, as well as the particular importance attributed to PLA material in the last decade.

## 7. Influence of FDM Process Conditions on Tensile Strength

Tensile testing of FDM-printed parts follows standardized protocols adapted from conventional polymer testing standards, primarily ISO 527 [82] and ASTM D638 [51]. The anisotropic nature of FDM parts and the influence of print orientation necessitate careful consideration of specimen preparation and testing procedures [34].

ISO 527 [82] and ASTM D638 [51] define multiple specimen geometries. The most commonly used specimens for FDM-printed parts include:

- *ISO 527 Type 1A*: Overall length 170 mm, gauge length 80 mm, width at ends 20 mm, width at narrow section 10 mm, thickness 4 mm.
- *ASTM D638 Type I*: Overall length 165 mm, gauge length 50 mm, width at ends 19 mm, width at narrow section 13 mm, thickness 3.2 mm.
- *ASTM D638 Type IV*: Overall length 115 mm, gauge length 25 mm, width at narrow section 6 mm, thickness 3.2 mm (commonly used for FDM parts due to smaller size).

Specimen preparation methods vary across studies. Direct printing (i.e., printing the final geometry directly) is more common due to simplicity and material efficiency, while machining from larger printed blocks can reduce surface roughness effects and edge defects [34]. Hozdić and Hasanagić [75] designed specimens in SolidWorks 2020 to ISO 527-2 [116] specifications and printed them on a Bambu Lab X1 Carbon Combo printer using Bambu Studio slicer software, demonstrating that careful integration of CAD design, slicing parameters, and testing protocol is essential for reproducibility.

Several challenges specific to FDM-printed specimens appear consistently across the literature:

- *Anisotropy*: Mechanical properties vary significantly with print orientation, necessitating testing in multiple orientations (XY, XZ, ZX) to fully characterize anisotropic behavior [34,71].
- *Surface roughness*: Layer lines create stress concentrations that may initiate premature failure. Some studies apply surface finishing (e.g., sanding, vapor smoothing) to reduce this effect [34].
- *Dimensional variability*: FDM parts exhibit greater dimensional variability than injection-molded parts, requiring measurement of actual specimen dimensions rather than sample dimensions [78].
- *Grip-induced failure*: Soft polymers (e.g., TPU) and brittle polymers (e.g., PLA) may fail at grips rather than in the gauge section; sandpaper or rubber inserts in grips can mitigate this issue.
- *Strain rate sensitivity*: Altahir et al. (2024) [117] demonstrated a linear rise in tensile and yield strength with increasing crosshead speed for FDM-printed PLA across a range of 0.8–20 mm/min, indicating that crosshead speed reporting is mandatory for cross-study comparisons.

Tensile testing provides multiple quantitative outputs, which include:

- *Ultimate tensile strength (UTS)*: Maximum stress sustained by the specimen during testing, calculated as maximum load divided by original cross-sectional area (MPa). UTS quantifies resistance to fracture under tensile loading.
- *Young’s modulus (elastic modulus)*: The slope of the initial linear portion of the stress–strain curve (GPa or MPa), representing material stiffness. Calculated from the elastic region, typically 0.05–0.25% strain.
- *Yield strength*: The stress at which the material begins to deform plastically, defined by the 0.2% offset method. Brittle polymers such as PLA may fracture before yielding [34].
- *Elongation at break*: The strain percentage at fracture, indicating material ductility.
- *Toughness*: The total energy absorbed until fracture, calculated as the area under the stress–strain curve (J).

Engineering stress–strain curves ($\sigma =F/A_{0}$ vs. $\varepsilon =\Delta L/L_{0}$) are the standard representations for comparative analyses, as they eliminate specimen geometry dependence. Force–displacement curves, while directly measured, cannot support cross-geometry or cross-study comparisons [34].

Layer height exhibits a complex, often non-linear relationship with tensile strength. Decreasing layer height generally increases tensile strength due to increased interlayer bonding area and reduced stress concentration at layer interfaces [34]. Reducing layer height from 0.3 mm to 0.1 mm increases PLA tensile strength by 15–25%. Syrlybayev et al., 2021 [34] and Gao et al. (2015) [73] identified similar trends for ABS and PETG, with optimal layer heights of 0.1–0.15 mm. The effect on Young’s modulus is less pronounced, with most studies reporting variations of less than 10% across typical layer height ranges [34]. Hsueh et al. (2021) [72] fixed layer height at 0.2 mm as an intermediate optimum for PLA and PETG, noting that this setting delivered consistent mechanical performance across both tension and compression loading modes.

*Infill density* demonstrates a strong positive correlation with tensile strength, modulus, and toughness. PLA tensile strength increases from approximately 22.49 MPa at 40% infill to 45.00 MPa at 100% infill, a 100.09% enhancement [75]. The relationship is non-linear, with diminishing returns above 70–80% infill, because tensile failure in FDM parts often initiates at interlayer interfaces rather than within the infill structure [73]. Young’s modulus scales more linearly with infill density, rising from approximately 595 MPa to 1051 MPa across the 40–100% range in PLA, because modulus reflects effective cross-sectional area resistance to deformation [75].

*Infill pattern* affects tensile properties through its influence on load distribution and failure modes. Honeycomb, triangular, and gyroid patterns generally provide superior strength-to-weight ratios compared to rectilinear patterns [34]. However, the effect of infill pattern on tensile strength, at similar infill density, is relatively modest. Typically, the tensile variation is less than 15% across common patterns because tensile failure is often dominated by interlayer bonding strength rather than infill architecture [34]. Ramírez-Prieto et al. [77] confirmed that infill pattern interacts with raster angle to govern fracture behavior: specimens with 45°/−45° raster angle tend to fail along the raster orientation due to shear stress development, regardless of infill pattern.

*Raster angle* is among the most influential parameters affecting tensile strength. Tensile strength maximizes at 0° (i.e., raster aligned with loading direction) and minimizes at 90° (i.e., perpendicular) [34]. Ramírez-Prieto et al. [77] reported that, for PLA+, parallel raster (0°/0°) yielded superior tensile strength, while crisscross (45°/−45°) yielded the lowest tensile value but the greatest ductility. For ABS, Kopar et al. [71] demonstrated that the 45°/−45° raster angle achieves the highest tensile performance compared to 0°/90° and other configurations, confirming material-specific optimal conditions that cannot be generalized across polymer types.

*Print orientation* fundamentally determines the relationship between loading direction and layer structure. The strength ratio between XY and Z orientations typically ranges from 1.5:1 to 3:1, depending on material and process parameters [34]. For ABS, Kopar et al. [71] confirmed that printing direction affects not only tensile strength but also residual stress accumulation, with higher residual stresses developing at lower printing speeds, which in turn influences the mechanical performance measured during tensile testing.

*Printing speed* exerts a complex, material-dependent effect. Kopar et al. [71] demonstrated for ABS that lower printing speeds achieved greater tensile strength, while higher speeds produced lower strength but greater ductility. The explanation involves a speed–temperature coupling: lower speeds allow greater thermal diffusion time at interlayer interfaces, improving bonding quality. However, the absolute magnitude of speed effects on tensile strength remains modest (<15% variation) compared to orientation and raster angle effects [34,71].

*Extrusion temperature* significantly affects interlayer bonding. Gao et al. [73] reported a 20% strength increase for PLA across a 190–220 °C range. Hsueh et al. [72] confirmed that both PLA and PETG tensile modulus and strength increase monotonically with printing temperature, attributing the effect to decreased polymer melt viscosity and enhanced layer fusion. At 180 °C, PLA has not fully reached its melting point, resulting in poor bonding and high interlayer porosity; stress–strain curves at this temperature diverge markedly from those at 200–220 °C [72].

*Moisture content* critically affects tensile properties in hygroscopic polymers. Sun et al. [76] documented a 30–40% strength reduction for Nylon, 20–25% for PETG, and 10–15% for PLA when relative humidity increased from 15% to 95%. Hozdić and Hasanagić [75] noted that their PLA filaments required storage in a humidity-controlled environment and use of filament dryers to maintain the mechanical consistency documented in the study, with manufacturer specifications indicating equilibrium water absorption below 0.3% by weight for PLA. Even short-term lubricant exposure (i.e., 30 days) reduced PLA tensile strength by 15.56%, illustrating the broader principle that chemical environments degrade tensile performance independently of process parameter optimization [75].

*Post-print annealing* can significantly improve tensile properties by relieving residual stresses, promoting crystallization in semi-crystalline polymers, and improving interlayer bonding [34]. Studies on PLA annealing report tensile strength increases of 10–30% and modulus increases of 20–40% when annealed at 80–100 °C for 1–4 h. Annealing effects are most pronounced for semi-crystalline polymers (e.g., PLA, PEEK, Nylon) and minimal for amorphous polymers (i.e., ABS, PC) [34].

Despite extensive research, significant contradictions exist across studies:

- *Polymer grade variability*: Alhuzaim [81] demonstrated substantial tensile strength variation among PLA sources under identical processing conditions. Hozdić and Hasanagić [75] observed PLA values closely matching manufacturer ISO 527 [82] specifications (45–49 MPa), while other studies report values as low as 22.49 MPa at 40% infill, illustrating that infill level and filament source jointly govern reported strength values and make cross-study comparisons unreliable without full material disclosure.
- *Machine calibration*: Printer architecture and calibration status affect dimensional accuracy, extrusion consistency, and thermal control, yet detailed calibration procedures rarely appear in publications [34,118]. Kopar et al. [71] demonstrated that residual stresses and deflections vary substantially with printer-specific thermal histories, suggesting machine-dependent variability extends beyond operator error.
- *Testing conditions*: Crosshead speed variations (1–50 mm/min) can affect measured tensile strength by 10–20% due to strain rate sensitivity of polymers [34]. Altahir et al. [117] confirmed a 30% increase in PLA UTS (from 40.41 MPa at 0.8 mm/min to 52.62 MPa at 20 mm/min) alongside a 43.5% increase in elastic modulus across the same range, a magnitude sufficient to explain many apparent contradictions in the literature.
- *Interaction effects*: Single-factor studies that ignore two-way interactions between layer height and extrusion temperature, and between infill density and raster angle, systematically misrepresent the influence of individual parameters [34,73].

*Underexplored variables*. The following potentially influential variables remain underexplored:

- *Filament diameter tolerance*: Few studies systematically investigate the effect of filament diameter variability on tensile properties [34].
- *Nozzle geometry*: Internal nozzle geometry (e.g., cone angle, land length, core heat) affects shear rate and polymer orientation during extrusion, yet researchers rarely investigate it [78,119].
- *Machine kinematics*: The effect of printer architecture (Cartesian, CoreXY, delta) on mechanical properties through motion dynamics and vibration lacks a systematic study [34].
- *Strain rate effects*: As demonstrated by Altahir et al. [117], crosshead speed can alter PLA UTS by up to 30%, yet most studies employ a single speed without justification.
- *Long-term aging*: Most studies test specimens within days of printing; Hozdić and Hasanagić [75] extended this to 30-day lubricant exposure, revealing 15.56% PLA strength loss, a result underscoring the need for longer-term degradation studies under realistic service conditions.
- *Filament color and additives*: Colorants and additives in commercial filaments may affect properties, but most studies use natural (uncolored) filaments or do not report color [78].

## 8. Influence of FDM Process Conditions on Compressive Strength

Compression testing of FDM-printed thermoplastic parts has received significantly less attention than tensile testing, despite its importance for structural supports, tooling, and load-bearing components. This section synthesizes current knowledge on compression testing methodologies, measurable outputs, test diagram interpretation, the influence of process parameters, contradictory findings, and underexplored variables.

Compression testing of FDM-printed parts follows protocols adapted from ISO 604 [120] and ASTM D695 [59]. The anisotropic nature of FDM parts and the influence of infill structure on compressive behavior require careful consideration of specimen preparation and testing procedures [34]. Aspect ratio selection governs failure mode: low aspect ratios (1:1) promote uniform compression and densification, while high aspect ratios (2:1 or greater) may promote buckling failure, particularly for low-infill specimens [34].

FDM-printed specimens pose specific challenges that must be considered before testing:

- *Infill collapse*: Low-infill specimens may exhibit progressive infill collapse rather than uniform compression, complicating stress–strain curve interpretation [34].
- *Buckling*: High aspect ratio specimens with low infill density may buckle rather than compress uniformly [34].
- *Barreling*: Friction between specimen and platens causes non-uniform stress distribution [34].
- *Densification*: Unlike injection-molded parts exhibiting clear yield and failure points, FDM-printed parts with infill often display progressive densification without a clear failure point, requiring compressive strength definition at 10% strain [34,72].

Compression testing provides multiple quantitative outputs, which include:

- *Compressive strength*: The maximum compressive stress sustained, or the stress at a specified deformation (typically 10% strain for infill parts) [34,72].
- *Compressive modulus*: The slope of the initial linear portion of the compressive stress–strain curve (GPa or MPa) [34].
- *Yield point*: The stress at which plastic deformation begins (0.2% offset method), though absent in low-infill specimens undergoing progressive collapse.
- *Densification behavior*: Three distinct regions characterize infill-bearing specimens: (1) linear elastic, (2) plateau corresponding to infill collapse, and (3) densification where stress increases rapidly as the infill structure fully collapses [34].

A critically important finding emerges across multiple studies: compressive stress in FDM-printed parts consistently exceeds tensile stress for any given strain, a tension–compression asymmetry driven by residual cooling stresses that accumulate during layer deposition [34,72]. Hsueh et al. [72] documented this asymmetry for both PLA and PETG across all tested printing temperatures, confirming that the compressive Young’s modulus exceeds the tensile Young’s modulus and that bending strength falls intermediate between tensile and compressive values.

*Layer height* exerts a less pronounced effect on compressive strength than on tensile strength. Studies report that layer height variations (0.1–0.3 mm) result in compressive strength variations below 15%, compared to 20–30% variations in tensile strength, because compressive loading is inherently less sensitive to interlayer bonding quality [34].

*Infill density* is the most influential parameter affecting compressive properties. Compressive strength and modulus increase approximately linearly with infill density, with 100% infill specimens exhibiting 3–5× higher compressive strength than 20% infill specimens [34]. Obaide and Saad et al. [121] demonstrated that compressive strength at 100% infill density was approximately ten times higher than at a low baseline infill density, with infill density identified as the dominant variable in ANOVAs, significantly outranking layer thickness and infill pattern in statistical contribution.

*Infill pattern* exerts a pronounced effect on compressive strength, greater than on tensile strength, due to the direct role of internal structure in resisting compressive loads. Yadav et al. [122] demonstrated that, among six infill patterns for PLA at 80% infill density, the Hilbert curve achieved 121.35 MPa, substantially exceeding rectilinear (78.88 MPa), line (73.84 MPa), honeycomb (62.56 MPa), Archimedean curves (70.07 MPa), and octagram spiral (60.01 MPa), a twofold range attributable to differences in load distribution efficiency. More recently, Patil et al. [123] investigated PETG+CF compression and found that trihexagon infill at 80% density achieved 39.16 MPa, while cubic infill at 40% density yielded only 11.52 MPa—a 3.4× difference confirming that pattern selection proves critical for compression optimization (see Table 2 and Table 3).

*Print orientation* significantly affects compressive properties through the relationship between loading direction and layer structure. Specimens loaded parallel to layers (XY orientation) exhibit higher compressive strength than those loaded perpendicular (Z orientation), mirroring tensile anisotropy [34]. Obaide et al. [121] confirmed that increasing infill density produces a direct increase in compressive strength across all orientation conditions.

*Printing temperature and speed* influence compressive properties through the same interlayer bonding mechanism as tensile properties. Hsueh et al. [72] documented that compression properties of both PLA and PETG increase with printing temperature, driven by decreased contact stress at line-to-line interfaces. Notably, PLA compressive properties increased with printing speed (due to rapid heat dissipation promoting fusion), while PETG compressive properties improved at lower speeds (due to the material’s slower heat dissipation characteristics), a material-specific inversion that mirrors the tensile behavior of these two polymers.

Despite the growing body of compression testing research, several contradictions and gaps persist:

- *Specimen geometry inconsistency*: Unlike tensile testing, where ASTM D638 [51] dominates, compression studies employ diverse specimen geometries (e.g., cubes, cylinders, prisms), aspect ratios (e.g., 1:1 to 2:1), and dimensions, making cross-study comparisons unreliable [34]. Obaide et al. [121] employed ASTM D695 [52] dimensions, while Yadav et al. [122] used the same standard but with different specimen heights, introducing buckling risk variability.
- *Failure criterion ambiguity*: Some studies define compressive strength at maximum stress, others at 10% strain (especially for low-infill specimens exhibiting progressive densification), and still others at first yield, introducing systematic measurement bias [34,72]. This lack of standardization prevents meaningful meta-analysis.
- *Pattern-specific effects underreported*: While Yadav et al. [122] documented a twofold compressive strength range across six infill patterns at constant 80% density (from 60.01 MPa for octagram spiral to 121.35 MPa for Hilbert curve), most studies examine only one or two patterns, systematically underestimating the design space available for compressive optimization.
- *Tension-compression asymmetry*: Hsueh et al. [72] demonstrated that compressive stress consistently exceeds tensile stress at equivalent strain due to residual cooling stresses, yet this fundamental asymmetry rarely informs material selection or structural design in the literature.
- *Temperature–speed coupling*: The observation that PLA compression properties improve with speed while PETG properties improve with slower speeds [72] suggests material-specific thermal dynamics govern compressive bonding quality, yet systematic studies mapping this relationship across the full material palette (e.g., ABS, Nylon, PEEK, TPU) remain absent.

*Underexplored variables in compression*. The following potentially influential variables remain underexplored:

- *Platens friction effects*: Barreling and non-uniform stress distribution due to platen friction rarely receive systematic investigation [34].
- *Cyclic compression behavior*: Most studies employ monotonic loading; fatigue and cyclic compression remain underexplored despite relevance for structural applications.
- *Multi-layer configurations*: Aboelella et al. [124] demonstrated that two-layer infill configurations outperform single-layer and four-layer designs, yet systematic exploration of layer stacking strategies for compression optimization is in its early stages.
- *Hybrid infill strategies*: Combining different infill patterns within a single specimen (e.g., dense shell + lightweight core) offers potential compressive optimization yet remains largely uninvestigated.

### 8.1. Synthesis and Future Directions

This literature review reveals a maturing but still fragmented understanding of FDM mechanical properties. Several overarching themes emerge:

- *Parameter hierarchy is established but material-specific*. Print orientation, layer height, infill density, raster angle, and extrusion temperature consistently rank as the top five influential parameters, yet their relative importance and interaction patterns vary dramatically by material [34,71,72,73]. Figure 5 illustrates this material-specific response landscape, showing that optimization strategies effective for PLA may prove counterproductive for TPU or PETG.
- *Environmental factors rival process parameters in magnitude*. Moisture content produces tensile strength reductions of 30–40% for Nylon [76], rivaling the effects of suboptimal process parameter selection. Yet environmental control protocols remain inconsistently reported and rarely integrated into optimization studies. Figure 3d demonstrates that hygroscopic polymers experience substantial performance degradation at humidity levels commonly encountered in laboratory and industrial settings.
- *Polymer grade variability undermines reproducibility*. The tensile strength variation observed by Alhuzaim [81] across PLA filament batches under identical conditions, alongside Hozdić and Hasanagić’s [75] demonstration that values range from 22.49 MPa to 45.00 MPa within a single infill density study, confirms that “PLA” as a material designation lacks sufficient specificity for rigorous comparative research. Future studies must report on manufacturer, lot number, and ideally molecular weight distribution.
- *Tension–compression asymmetry is universal but underutilized*. The systematic finding that compressive properties exceed tensile properties [72] has profound implications for structural design, yet current design guidelines rarely exploit this asymmetry.
- *Infill pattern selection is under-optimized*. The twofold compressive strength range across infill patterns at constant density [123,124] indicates that pattern optimization offers equivalent performance gains to density optimization, yet pattern receives far less research attention. The Hilbert curve, achieving 121.35 MPa compared to honeycomb’s 62.56 MPa at identical 80% infill density, demonstrates that internal architecture warrants equal consideration alongside density in structural design.

### 8.2. Future Research

Adopt standardized material characterization protocols, including molecular weight, moisture content, and thermal history reporting across all publications.

Employ full-factorial or response surface designs to capture parameter interactions systematically, building on the interaction analysis demonstrated by Kopar et al. and Gao et al. [71,73].

Extend testing beyond quasi-static monotonic loading to include cyclic fatigue, creep, impact, and environmental degradation under realistic service conditions extending beyond 30 days.

Develop validated multi-scale models coupling process simulation (e.g., thermal history, residual stress accumulation) with mechanical performance prediction, following the FEA approach demonstrated by [71].

Investigate hybrid and functionally graded structures exploiting spatial variation in process parameters and infill architecture—building on the promising results of Aboelella et al. [124] for multi-layer infill configurations.

Systematically map the complete design space for infill patterns in compression, expanding beyond the six patterns investigated by Yadav et al. [123] to include triply periodic minimal surfaces, bio-inspired structures, and computationally optimized topologies.

## 9. Influence of FDM Process Conditions on Torsional Strength

**Figure 8:**
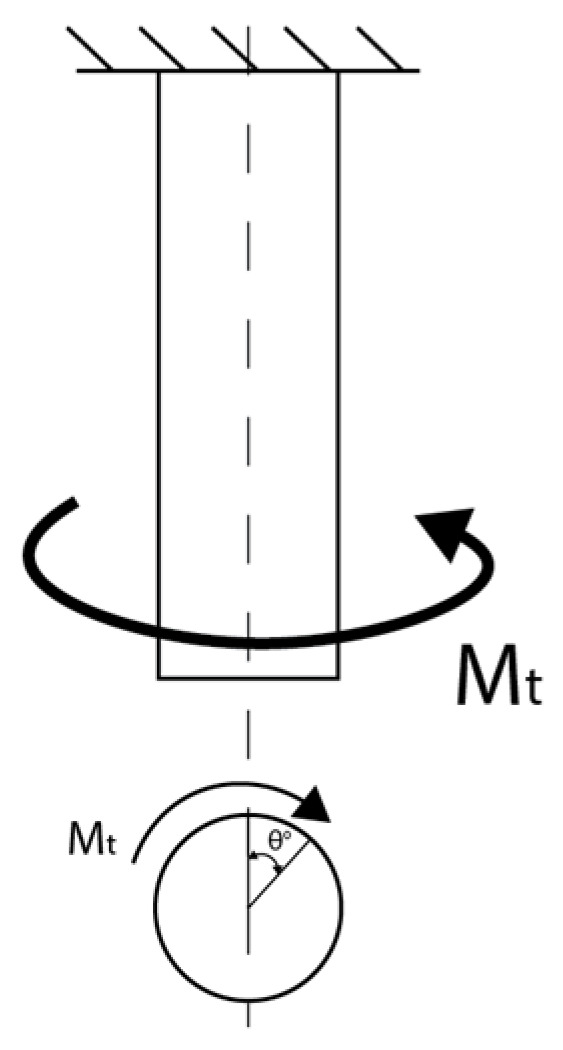
Torsion test on a cylindrical sample (adapted from [127]).

The way in which the torsion test occurs is presented in Figure 8; the testing machine records the applied torque *T* and the angle of twist *θ*° to create the representative torque–twist diagram (Figure 10). Generally, there are no specific standards about additively manufactured specimens for torsion tests. The most appropriate standard refers to plastics in general and the torsion test for plastics, which identifies the specimen as a full-solid object. Researchers use the standards that seem more appropriate, for example, ISO 18338 [125], which was initially designated for metal pieces, or ASTM E143 [126], for shear modulus [127,128,129].

The equation to find out the torsion strength (maximum shear stress) *τ_max_*, where *M_t_* means the maximum breaking torque and *J* is the polar moment of inertia, is the following:

(1) $$\tau_{max}=\frac{M_{t}\cdot r}{J},$$

Unfortunately, in additive manufacturing, there is no such thing as a full-solid sample, because printing involves a sample made of layers and a lot of air gaps.

**Figure 9:**
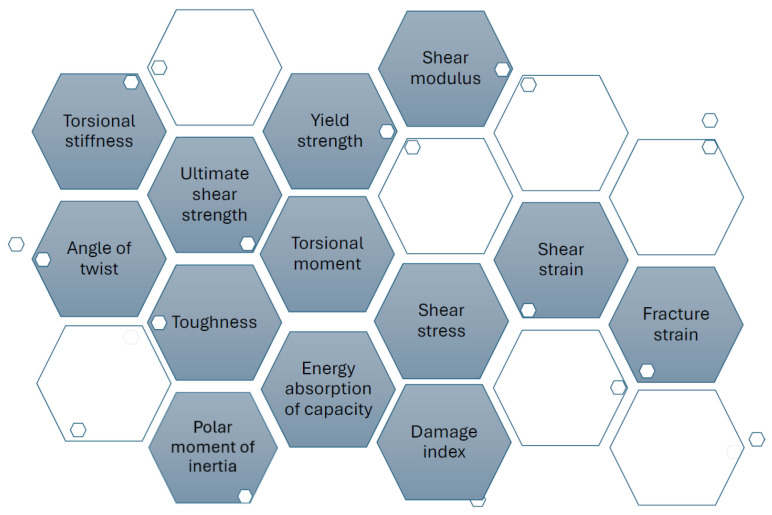
Characteristic parameters for the torsion test.

Two types of monitoring have been identified for the torsion test, the first is the classical method, in which the machine records torque and angle of the twist *θ*, and the second is a modern approach, known as digital image correlation (DIC), that registers and follows the pixels of the image of the experimental sample, so every movement is recorded in 2D space [130,131]. During the torsion test, we can get more parameters about material properties, presented in Figure 9. These metrics help scientists to be more specific about different material or process states.

**Figure 10:**
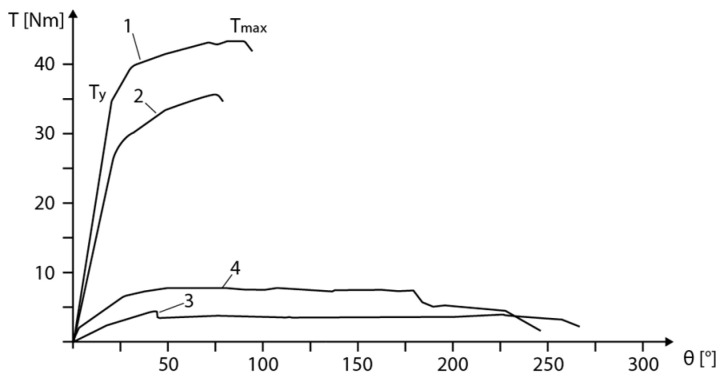
Torsional behavior of FDM printed polymers: 1—PLA (grid infill) (adapted from [132]), 2—PLA (lines infill) (adapted from [132]), 3—ABS ±45° raster angle) (adapted from [127]), 4—PLA ±45° raster angle) (adapted from [127]).

Figure 10 represents the torsional behavior within two experimental studies, that includes the usual segments for a mechanical test, such as an elastic region and plastic deformation region (after the yield point *T_y_*), and then the samples reach the peak torque *T_max_*, followed by breaking.

Curves 1 and 2 represent ductile behavior and the authors Kaya and Yaman state that the external wall had a brittle fracture, while infill regions experienced a ductile behavior, because of the infill pattern (grid, lines) that helps redistribute stress [132]. A characteristic of brittle behavior to have a fracture of 45° was observed by Sadaghian et al. as was the occurrence of residual strength (curves 3 and 4), that appears when interlayer bonding is weaker than layer resistance and breaks first [127]. Curves 1 and 2 tend to have a much higher torsional moment, as the samples were printed with a vertical build orientation, while samples 3 and 4 were printed using flat orientation, along the axis of the samples that coincide with torsional axis [127,132].

The most important parameter for the FDM process is considered infill density. The 100% infill density increases the torque and stiffness to the maximum value, because it reduces the number of air gaps that weaken the structure [129,130]. The Torres et al. trials show that increasing infill the level from 20% to 100% leads to an increase in ultimate shear strength with 15–25 MPa [129]. Also, research by Sharma et al. for a potential PLA substitute of a bone concluded that increasing the infill density from 20% to 100% means increasing torque from 1400 N·mm to above 2400 N·mm [133].

The recommendation is to use a ±45° raster angle and not 0°/90°, because it offers better torque strength due to the more efficient shear stress distribution along the filament [134]. In Sadaghian et al.’s experiment, all 15 samples with a ±45° raster angle reached a higher torsional load, for example, for PETG it is higher with approx. 2 Nm (55%) than that of the PETG printed with 0°/90° raster angle, for ASA and ABS the differences are 55% and 29% [127].

Studied papers confirmed that a small layer height contributes to a stronger interlayer bonding and increases the sample density which improves resistance [129]. For PLA samples with the same 20% infill density, Torres et al. obtained a decrease in shear stress from 28 MPa to 20 MPa for an increase in layer thickness from 0.1 to 0.3 mm [129]. In Sharma et al.’s experiment, an increase in layer height from 0.1 to 0.5 mm resulted in an average torque reduction of 38.43% [133].

It was observed in the preliminary testing of the research of Torres et al. that better torque resistance is provided by samples printed with horizontal build orientation and this idea is supported by Sharma et al. when speaking about PLA material, as samples with other orientations tend to have premature delamination between layers [129,133].

The best identified infill pattern configuration from unit cell models: trunch octa dense, trunch octa light and body diagonals with nodes (BDN), resulted in BDN with a torque *T* = 1.33 Nm at a twisted angle of 34° for PLA samples [130]. The comparison between the infill patterns of lines, gyroid, grid and random lines for PLA samples determined the following conclusions: the grid and random lines reached the highest torque, above 40 Nm; but grid and lines had a bigger angle of twist, the maximum being 80° and 67°, respectively [132].

The thicker the wall is, the higher torsion resistance is for PLA samples in Sharma et al.’s tests. When increasing the wall thickness from 0.4 to 1.2 mm, torque improved by 28.91%, reaching values up to 2.4 Nm [133].

Print speed affects torsion strength negatively, because it creates weaker interlayer bonding, increases the number of air gaps, and increases the risk of low material feed. In the tests conducted by Sharma et al. a range of printing speeds of 20–100 mm/s were used and results showed a decrease in torque by 32% [133].

Effects of the heat treatment of the samples could increase the torque resistance by a small amount, but generally, it is considered that this also leads to a brittle behavior because heating generates gas bubbles, which represent a risk for crack initiation. For instance, Torres et al. used a Taguchi model of testing with 0 min, 5 min and 20 min healing time parameters at 100 °C for PLA samples and the results showed that the heat treatment has influence of 11% on yield strength, 14% on the ultimate shear strength, 22% on shear modulus and 41% influence on fracture strain, that makes the material more brittle [129].

Nozzle temperature enhances filament fusion, which increases shear strength between layers. Ali et al. mentions that fracture toughness of the material was increased by up to 95% by increasing the printing temperature for PLA (200–210 °C)–Nylon (240–250 °C) samples, but there is an observation that the difference between the ambient environment and the printing temperature could cause internal stress [134].

## 10. Influence of FDM Process Conditions on Bending Strength

**Figure 11:**
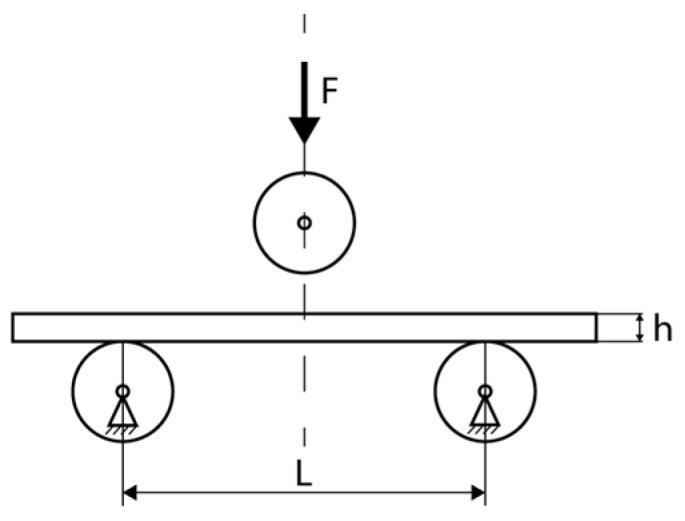
Bending test schematic representation (adapted from [138]).

The usual method to determine the bending strength of polymeric materials is standardized by ASTM D790 [53] and ISO 178 [135], as shown in Figure 11, using a three-point loading system [136]. The equipment used to identify the maximum load force is usually a universal testing machine (UTM) [137].

Bending strength (*σ_B_*) can be determined using Equation (9), where *F* represents the maximum load force, *L* is the distance between the support points, *b* is the width of the sample, and *h* is the thickness of the sample [139]:

(2) $$\sigma_{B}=\frac{3\cdot F\cdot L}{2\cdot b\cdot h^{2}}$$

Standard ISO 178 [135] and standard ASTM D790 [53] have recommendations about the dimensions used for plastics to determine bending properties that are used by researchers [136,138] but there are some conditions when the dimensions were adapted, such as expensive materials (e.g., PEEK), special medical conditions (for example, bones), or industrial conditions [137,140,141,142].

**Figure 12:**
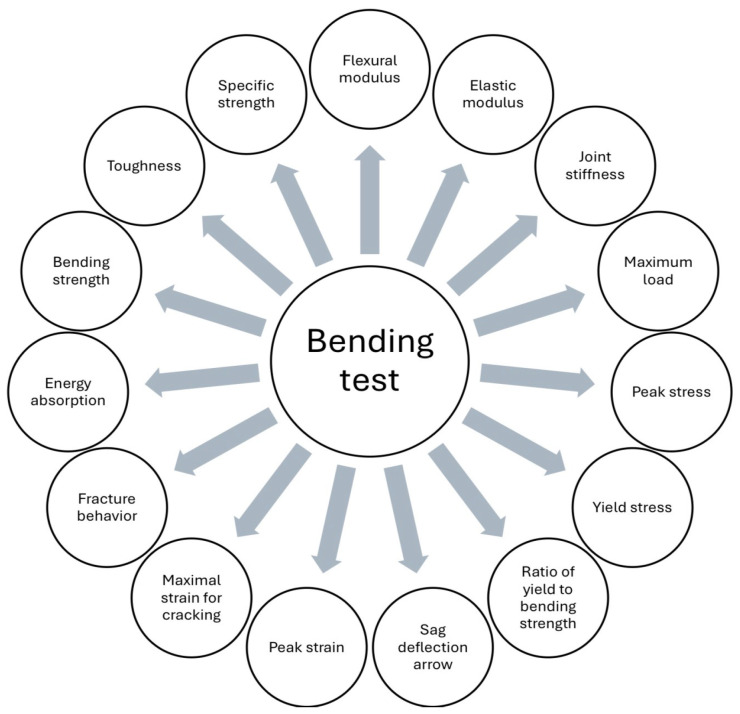
Representation of parameters identified due to the bending test.

In some cases, researchers use the four-point loading system, which is like the three-point loading system; there are two support points and two loading points, which need four rolls. This system is used for complex structures, such as joints, to observe other phenomena such as shear or peel stress [140]. The bending test determines several material-specific coefficients or information about the structure of the sample (Figure 12).

**Figure 13:**
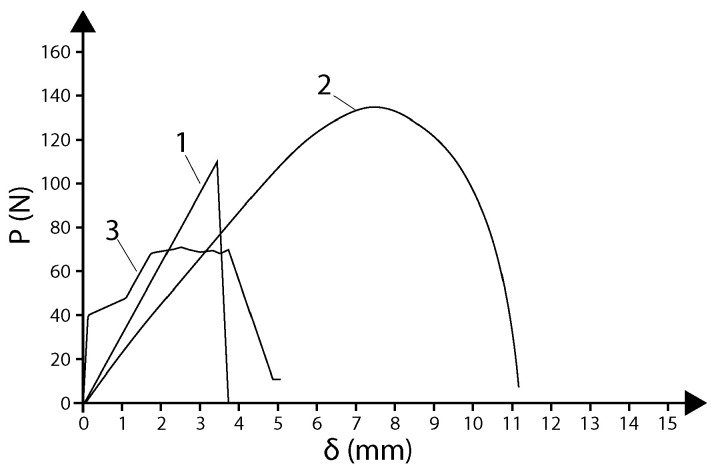
Load–displacement curves for FDM samples (1—PETG, adapted from [136], 2—PLA, adapted from [138], 3—PLA adapted from [145]).

The generic diagram that usually results after the bending test is a load (*P*)–displacement (*δ*) curve (*P* - *δ*) [140], as shown in Figure 13, where three types of behavior of a fracture are represented. The first curve (1) is characteristic of brittle failure, found in PETG samples, PEEK, and PLA [136,137,142]. Also, brittle behavior was observed in PEEK material in the conditions of 0.3 mm layer thickness and printing temperature of 445–525 °C that could not melt the layer totally because of the thickness and the vertical direction of printing [137].

The second curve describes the ductile behavior. In scientific literature, it was observed that PETG, PLA, and locally pre-heated ABS are good examples of ductile failure behavior in bending tests [136,141,143].

The third curve is the representation of interlayer debonding, where every layer has the behavior of an individual structure because of poor adherence between the printed layers. This fracture behavior happens in the case where PEEK material samples are printed vertically at low temperatures [137], print properties are not properly selected or because of inconvenient ambient conditions [144].

Materials are generally classified by being brittle or ductile, but the samples do not always have the same fracture behavior because of some process or structure parameters that make a difference, even though the material is the same. These factors will be discussed next.

Infill density is one of the most important factors that influence the bending strength [146]. Experimental research confirmed that a higher infill density leads to a higher bending strength and stiffness. Neccaroğlu et al.’s (2025) study highlights an over 4 times increase in the bending force, from 6 N to 27 N, in the case of ABS samples with an infill density of 40% and 100% [147]. For PETG samples, it was observed that decreases in infill density from 100% to 75% and 75% to 50% caused decreases in strength of 5–6% and 4.5–9.5%, respectively [146].

Previous studies about different infill patterns have shown that the triangular infill pattern has a higher bending strength, because in the interior of the sample, it acts like lattice beams, effectively absorbing compressive loads. The maximum load, in the Li et al. (2025) experiment, for a PLA triangle infill pattern was 9.34 N, while for wiggle, grid and full-honeycomb infill patterns they were 7.42 N, 6.77 N, and 6.39 N respectively [141]. The concentric and rectilinear infill patterns also demonstrate good bending resistance if the layers have the same orientation as the loading direction or high fill density [139]. Samples of PLA with a concentric infill pattern reached a bending force of 135 N for 100% infill density, with 17 N more than the triangle infill pattern in PLA samples [138]. The best result of bending strength for PLA samples between the rectangular, triangle and honeycomb infill patterns was 61 MPa for the rectangular pattern with 80% infill density, but also for 30% infill density the best result was 40 MPa by the honeycomb infill pattern which could be a solution in cases of low infill density models [139]. Another good infill pattern for bending strength is the wiggle due to its zig-zag configuration, which generally enhances the mechanical properties and dissipates more energy. Even though Li et al. obtained a value of 9.341 N for the triangle infill pattern, wiggle was noted as the second most resistant pattern with a bending force of 7.421 N, over grid and full honeycomb [141].

Another important parameter is layer height. There are observations that the decrease in layer thickness contributed to the increase in bending strength because the extrusion pressure of a vertical nozzle increases and creates a dense layer, with better interlayer adhesion, and the air gaps are minimized [147]. As an example, Wang et al.’s paper represents a decrease of about 6 MPa of bending strength for every increase in layer height of 0.1 mm for PLA samples [142]. Also, the best result for flexural strength for PETG samples was 70 MPa with a layer height of 0.1, with 4 MPa more than for 0.15 mm and 0.2 mm layer heights [146]. However, other researchers observed that a thicker layer means an increase in flexural strength [144]. That could be because the thicker layers mean fewer imperfect layers, less shear stress, and the risk of failure decreases [34]. In the context of 0.2 mm, 0.3 mm and 0.4 mm layer heights, the 0.4 thickness represented the best result of 49 MPa for flexural strength for PLA samples, while the best result of 35 MPa for ABS samples was for a 0.3 mm thickness [144]. That means that the layer thickness needs to be selected based on experimental practices on specific material, and attention needs to be paid to the combination of selected parameters.

Nozzle temperature is a parameter that generally enhances the FDM process, but there is a limit of temperature for every specific material, and exceeding this limit leads to polymer degradation. For example, Fountas et al. had the best result for flexural strength of 67.6 MPa for the highest nozzle temperature of 230° [148]. Kumar et al.’s research also demonstrated that an increase in nozzle temperature from 210 °C to 230 °C contributed to a 10% change in flexural strength for PLA samples [145]. This is because, at a higher temperature, the material has a lower viscosity, which means a better interlayer connection and fewer air gaps [145]. Wang et al. suggest to not exceed the nozzle temperature of 300 °C for PLA samples, keeping it in the range of 230 °C and 240 °C, because of material degradation [142].

In the literature, it is mentioned that a high printing speed leads to deterioration of material properties. It is because the high speeds contribute to decreasing the layer thickness due to insufficient time for melting in the nozzle, under-extrusion phenomena, and increased porosity, increasing the elongation that contributes to decreasing the material strength [145,149,150]. Kumar demonstrated that, for PLA samples, when increasing the printing speed from 25 mm/s to 55 mm/s, the mean of porosity increased from 4.4 to 11.3, having the most influence compared to nozzle temperature and raster width [145]. Gunay et al. also demonstrate the negative influence of the speed, and the best result for bending strength of 65 MPa was for the minimum printing speed of 30 mm/s [149]. There is a different argument, when setting a specific speed range and depending on the level of dissipation of the heat, that increasing the printing speed for PLA material could cause a faster heat dissipation and improve material properties, for example, increasing the printing speed for PLA from 35 mm/s to 45 mm/s increased the bending strength by 5 MPa at a printing temperature of 200 °C, while increasing the printing speed for PETG from 25 mm/s to 35 mm/s meant decreasing the bending strength by 5 MPa at 225 °C [72].

On the other hand, a thicker shell means better bending resistance, because the maximum stress in case of bending is on the external layers, and the thicker the shell is, the larger the load-bearing area. An increase from 1.2 mm to 1.8 mm of shell thickness meant an increase in bending strength from 69 MPa to 84 MPa for PLA samples [146]. The idea that shell thickness positively influences bending strength resulted in other experiences, whereby, when increasing the number of top layers of the shell, the bending strength increased by 4–6% [151].

Also, a technique was investigated to increase the bending load resistance. This solution refers to in-process laser heating or local pre-heating, which increases the interlayer bonding and bending strength. Ravi et al.’s results prove that maximum bending load for normal FDM printing was 80 N with a displacement of 0.8 mm, while samples with in-process laser printing resisted a 130 N bending load for 1.3 mm displacement. Consequently, strength improved by over 60% and elasticity improved by almost 100% [143].

## 11. Influence of FDM Process Conditions on the Impact Resistance of Polymeric Materials

**Figure 14:**
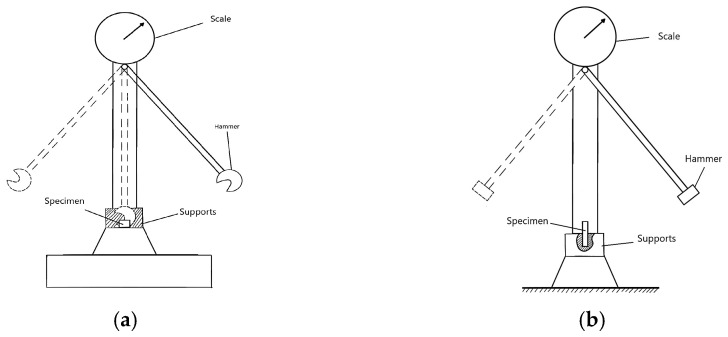
Mechanical test of the (**a**) Charpy and (**b**) Izod types.

For polymeric components fabricated via FDM, impact strength is of critical significance because the process-specific layered architecture induces anisotropic mechanical behavior, where the interfaces between deposited tracks often serve as preferential sites for crack initiation and propagation [152]. The quantitative assessment of impact resistance in polymers is typically conducted using Charpy or Izod pendulum tests, as standardized by ASTM D256 [55] or ISO 179 [153]. In these procedures, the specimen is struck by a pendulum of known potential energy. The energy absorbed during the fracture event is calculated as the difference between the initial potential energy and the residual energy of the pendulum post-impact. By normalizing this absorbed energy to the specimen’s cross-sectional area, the impact toughness is determined and expressed in kJ/m^2^. A schematic representation of these experimental configurations is illustrated in Figure 14.

In these cases, other relevant mechanical quantities may also be determined, such as the impact force, displacement at fracture, or the energy associated with crack initiation and propagation [154].

The mechanical integrity of components fabricated via FDM is governed by a complex interplay of processing parameters, including infill density, deposition speed, layer height, extrusion width, and liquefier temperature. These variables directly dictate the internal mesostructure of the part and the resultant interlayer bond strength [155]. Such factors critically modulate molecular diffusion across successive layers and the formation of interfilament porosity, leading to pronounced variations in the material’s constitutive behavior [156].

Furthermore, investigations carried out at the “Gheorghe Asachi” Technical University of Iasi have demonstrated that nozzle geometry and localized thermal gradients significantly influence the rheological stability of the extrusion process, thereby determining the final mechanical performance of the printed specimens [157].

The data presented in Table 4 show that the technological parameters significantly influence the impact resistance of polymeric materials manufactured by FDM. Among these, infill density has the strongest effect: increasing the infill reduces porosity and improves structural continuity, which leads to higher impact resistance values. Hasan et al. report an increase from 14.8 to 30.5 kJ/m^2^ for infill values ranging from 20% to 100% [152], Banić et al. report values between 16.9 and 32.0 kJ/m^2^ over the same range [158], while Tunçel indicates higher values, between 34.66 and 38.54 kJ/m^2^, for infill values between 40% and 100% [156], the differences being attributable to distinct experimental conditions.

**Figure 15:**
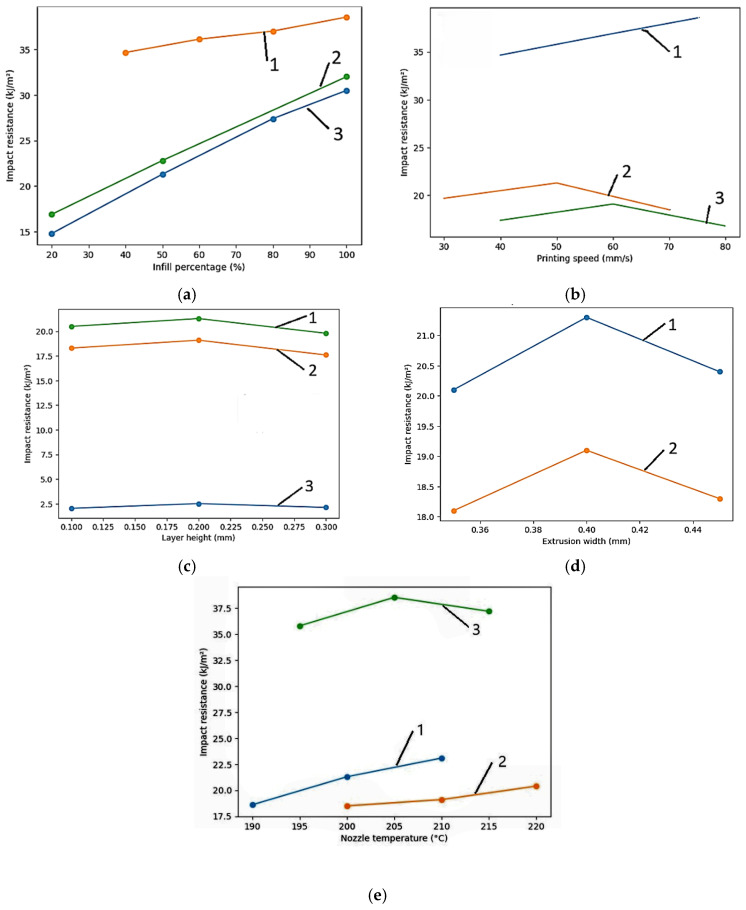
Impact resistance as influenced by: (**a**) infill density (1—[152]—PLA, 2—[156]—PLA, 3—[158]—PLA); (**b**) printing speed (1—[156]—PLA, 2—[152]—PLA, 3—[155]—PLA); (**c**) layer height (1—[159]—PLA, 2—[155]—PLA, 3—[152]—PLA); (**d**) extrusion width (1—[152]—PLA, 2—[155]—PLA); and (**e**) nozzle temperature (1—[152]—PLA, 2—[155]—PLA, 3—[156]—PLA).

The influence of infill density is illustrated in Figure 15a, where a general increase in impact resistance can be observed with increasing infill, as a result of reduced internal porosity and improved structural continuity of the material.

The influence of printing speed on impact resistance is presented in Figure 15b and is non-uniform in nature, with the literature occasionally indicating divergent trends. Tunçel reports an increase in impact resistance with increasing printing speed [156], whereas Hasan et al. identify a maximum at a printing speed of 50 mm/s, followed by a decrease at higher speeds [152], a trend also confirmed by Beníček [155]. These differences may be explained by the reduction in thermal contact time between layers at higher speeds, which limits molecular diffusion, weakens interlayer bonding, and reduces mechanical resistance.

The influence of layer thickness is presented in Figure 15c. The data indicate a non-linear response of impact resistance, with optimal values generally around 0.2 mm. Hasan et al. report an increase from 20.5 kJ/m^2^ (0.1 mm) to 21.3 kJ/m^2^ (0.2 mm), followed by a decrease to 19.8 kJ/m^2^ (0.3 mm) [152], a trend also confirmed by Beníček et al. [155]. Milovanović et al., however, report much lower values, of only 2.04–2.52 kJ/m^2^, suggesting either different experimental conditions or a distinct geometry of the specimens used [159].

Extrusion width has a smaller influence compared with the other analyzed parameters. Hasan et al. indicate a maximum impact resistance of 21.3 kJ/m^2^ for a width of 0.40 mm, compared with 20.1 kJ/m^2^ at 0.35 mm and a slight decrease at 0.45 mm [152]. An optimal value promotes adequate lateral coverage of the deposited roads, material cohesion, and a more uniform distribution of internal stresses. This influence is presented in Figure 15d.

The influence of nozzle temperature on impact resistance is presented in Figure 15e. In general, increasing the extrusion temperature improves impact resistance by promoting molecular diffusion and interlayer bonding. Hasan et al. highlight an increase in impact resistance from 18.6 kJ/m^2^ at a temperature of 190 °C to 23.1 kJ/m^2^ at 210 °C [152]. Tunçel et al. indicate an optimum around 205 °C, followed by a slight decrease, which may be explained by incipient thermal degradation or process instability at excessively high temperatures [156].

The specialized literature shows that impact resistance is also influenced by other factors, such as print orientation, infill pattern, number of outer layers, nozzle diameter, and extrusion conditions, which can modify the internal structure, stress distribution, and the quality of filament deposition [156,158].

## 12. Influence of FDM Process Conditions on the Fatigue Resistance of Polymer Materials

As in the case of metallic materials, polymeric materials are often subjected to variable loads of tension, compression, torsion, bending, etc. The variable nature of the loading is given by the evolution of the direction over time, together with a change or no change in the magnitude of the loading. Under such conditions, it is possible for the material to undergo a “fatigue” phenomenon and to fail before reaching the maximum stress value that would cause, under the same type of loading but static, the failure of the material (σ < σ_max_). Fatigue constitutes the primary cause of failure for parts in motion. The moment of failure of parts subjected to variable loads depends on the material’s property of responding to such loads, a property materialized through the material’s fatigue strength. This is a characteristic that must be considered when the part is subjected to loading.

A cycle of variable loading in which the stress varies between a maximum value (σ_max_) and a minimum value (σ_min_) is characterized by the value of the amplitude (σ_max_ − σ_min_)/2, the value of the mean stress (σ_max_ + σ_min_)/2, and, perhaps most importantly, the asymmetry coefficient R, defined as:

(3) $$R=\frac{\sigma_{min}}{\sigma_{max}}.$$

**Figure 16:**
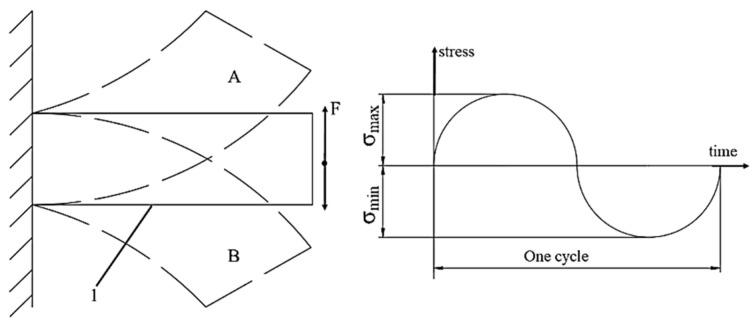
Schematic diagram of a variable alternating bending test with a symmetrical alternating cycle, characterized by σ_max_ = σ_min_ and R = −1.

An example of a variable alternating bending test with a symmetrical alternating cycle is shown in Figure 16. It can be observed that, under the action of the variable force *F*, during one cycle, specimen 1, which is fixed at one end, reaches the maximum top position A and the minimum bottom position B. These positions are reached repeatedly, while the stress oscillates between the maximum (σ_max_) and minimum (σ_min_) values, as a result of cyclic loading with force *F* between certain minimum and maximum values.

The definition of fatigue strength for polymeric materials is done in the same way as for metallic materials. The determination of fatigue strength is carried out experimentally. Given that the results obtained for the parameters characterizing fatigue strength in tests using a symmetrical alternating cycle are superior to those obtained using other variable cycles (oscillating, pulsating, alternating), this type of test is used, including for parts made of polymeric materials, to determine fatigue strength. To express fatigue strength (σ_R_), the maximum stress is determined, at which the material withstands, without deterioration, an unlimited number of oscillating cycles characterized by R = −1, which defines a symmetrical alternating cycle.

Fatigue tests, both for metallic materials and polymeric materials, are carried out on specialized machines and are distinguished according to the type of loading. Thus, tests are encountered for axial loading, alternating bending tests, rotating bending tests, torsion tests, as well as complex tests such as combined bending and torsion tests or biaxial and triaxial tests [160].

**Figure 17:**
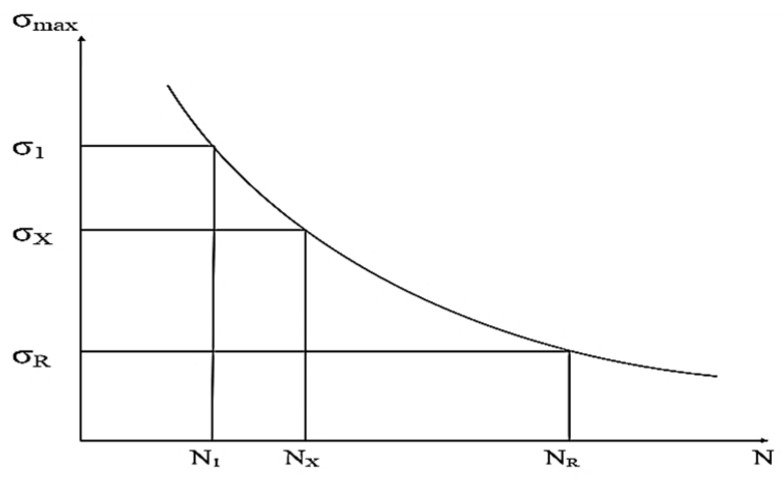
Exemplification of the method for plotting the characteristic fatigue curve (*Wöhler curve*). (adapted from [160]).

The curve resulting from experimental tests, carried out through variable tension, compression, torsion, and bending loads on material specimens using dedicated machines for determining fatigue strength, is called *the Wöhler curve* (in honor of the German engineer of that name who first created such curves). The plotting of these graphs is done in coordinates of applied stress (*σmax*) versus number of cycles to failure (N, usually on a logarithmic scale) (Figure 17), by successively testing a number (between 6 and 10) of identical specimens. It can be observed that, initially, a variable test is performed, characterized by a stress value *σ_max_* = *σ*_1_ with very high values (usually about two-thirds of the corresponding static strength), resulting in a relatively small number of cycles N_1_ to failure. The testing continues with further tests at progressively lower stress values σ_x_, thus resulting in an increasingly larger number of cycles to failure N_x_. This leads to a maximum stress value, σ_R_, which represents the fatigue limit at which the material does not fail even at an infinite number of loading cycles N_R_ (in reality, this number is up to 10^10^, and for polymeric materials even lower).

Plotting the characteristic fatigue curve for polymeric materials has been the concern of many researchers, given their increasingly frequent use in various industries. Few studies present the mechanical behavior of polymers, and it is accepted that, in the failure mechanism of polymers due to fatigue, an important place is held, alongside crack initiation (mechanical fatigue), by material softening accompanied by stiffness reduction caused by heating during loading (thermal fatigue) [161,162]. The study of the fatigue behavior of polymers and the determination of specific characteristics is carried out on specialized machines, through experimental tests, with specific standards developed by the American Society for Testing and Materials (ASTM) and the International Organization for Standardization (ISO) being known. Thus, ASTM D7774 [163] standardizes the method for determining the fatigue properties of plastics in bending, applicable to rigid and semi-rigid plastics, while ISO 13003 [164] addresses the same subject for fatigue testing of fiber-reinforced plastic composites under conditions of constant amplitude and constant frequency cyclic loading. ASTM D3479/D3479M-19 [165] clarifies the method for determining fatigue strength under tensile loading, and ASTM D7791-17 [166] clarifies fatigue testing in relation to the specimen surface condition.

The fatigue strength of polymeric materials obtained through material extrusion additive manufacturing (FDM) is influenced by the material anisotropy resulting from the material deposition method (raster orientation, layer height, bead width, printing speed, etc.) [71,73].

For common polymers such as PLA and ABS, experimental research has led to obtaining relationships of dependence of fatigue stress on the number of cycles to failure, of the Basquin model type [167]:

σ_x_ = A(N)^b^, (4)

where N is the number of cycles and A and B are determined experimentally. These relationships differ depending on the specific conditions of each study, and the results are different and sometimes relatively difficult to compare due to the characteristics of the testing method used. Furthermore, the value of applied stress often differs, which means that estimations are only indicative in terms of trend, not in absolute values. Values of the constants A and B presented in some works are indicated in Table 5.

Recent research also indicates the influence of temperature during loading [172], so a summary of these dependencies is presented in Table 6.

It can be stated that the fatigue strength of polymeric materials obtained via FDM is not yet fully understood. The influences are numerous, and absolute values are not fully accepted. Any type of material and any type of testing can be subjects of study.

## 13. Influence of FDM Process Conditions on the Hardness of Polymer Materials

The determination of the hardness of polymeric materials is primarily carried out by measuring the resistance to penetration of a hard body (indenter) into the surface of the material. Unlike metals, polymers exhibit a pronounced viscoelastic behavior, which means that the hardness value depends significantly on the load application time, temperature, and loading rate [189]. The most commonly used methods are Shore A (for soft polymers, such as elastomers or thermoplastic polyurethane) and Shore D (for rigid polymers, such as ABS or polycarbonate), both being regulated by the ISO 868 standard [190]. The device used, called a durometer, measures the penetration depth under the action of a calibrated spring, with the value being read on a scale from 0 to 100 units.

Another essential method for technical polymers and composites is Rockwell hardness, regulated by the ISO 2039-2 standard [191]. This method is based on measuring the difference in indentation depth between a preliminary load and a major load, typically using a hardened steel ball as the indenter. This method is preferred when it is desired to eliminate errors caused by surface irregularities or the immediate elastic recovery of the polymer. Furthermore, the ball indentation method (ISO 2039-1) [192] is widely used for determining surface hardness, calculating the ratio between the applied force and the area of the indentation surface after a period of 30 s of maintaining the load [193].

In advanced research, nanoindentation or instrumented hardness testing is used, which allows continuous monitoring of the indenter displacement with nanometric resolution [193]. This technique is crucial for polymeric materials obtained via FDM because it allows hardness testing on individual filaments or at the interface between layers (bonding zones). In addition to the nominal hardness value, this method can provide valuable data on the elastic modulus (indentation modulus) and energy absorption capacity, offering a complete picture of how the layered structure withstands localized mechanical loading [193].

By analyzing the indentation curves, the creep phenomenon can also be evaluated, observing how the penetration depth changes under a constant load maintained over the long term [189]. This characteristic is vital for 3D-printed components that will be used in mechanical assemblies under stress, as it indicates the risk of permanent surface deformation over time, even at loads below the material’s yield strength.

A schematic graphical representation of the instrumented hardness test highlights the relationship between the applied force (F) and the penetration depth (h). The resulting generic diagram shows an ascending loading curve, where the material undergoes simultaneous elastic and plastic deformations, followed by an unloading curve [193]. In the case of polymers, the unloading curve is not a straight line but a curved one, demonstrating the material’s partial elastic recovery. The difference between the maximum depth under load (h_max_) and the final depth after force removal (h_f_) indicates the degree of permanent deformation [193].

A generic example of a diagram resulting from the test shows that, for polymers such as PLA, the slope of the unloading curve is much smoother than in the case of metals, which indicates a much lower modulus of elasticity. Also, if the maximum load is maintained for a period, a horizontal segment (plateau) appears on the diagram, marking the increase in depth under constant force, a phenomenon specific to the viscoelastic behavior of plastics [189].

In the layered deposition (FDM) manufacturing process, the hardness of the finished part does not depend solely on the intrinsic properties of the material but is a structural property critically influenced by the printing settings. Because FDM parts are, by their nature, assemblies of molten polymer “lines” with microscopic voids between them, the hardness measured on the outer surface reflects the degree of cohesion and the density of these layers [193]. The influence of key printing parameters on hardness is summarized in Table 7.

## 14. Influence of FDM Process Conditions on the Vibration-Damping Capacity of Polymeric Materials

In the case of parts manufactured by FDM, this property is of particular interest, since the layered structure, residual porosity, quality of interlayer bonding, and filament orientation lead to anisotropic mechanical behavior, different from that of dense materials obtained by conventional methods [195,196].

In literature, damping capacity is determined by several experimental methods. Experimental modal analysis and impact tests on cantilever beams allow the determination of the modal damping ratio, *ζ*, using the half-power bandwidth method or the logarithmic decrement of amplitude [195,197]. Laser Doppler vibrometry provides a non-contact method, with high spatial resolution, for identifying natural frequencies, modal amplitudes, and damping ratios [196]. In addition, dynamic mechanical analysis is frequently used for the viscoelastic characterization of polymers, through the determination of the storage modulus, *E*′, the loss modulus, *E*″, and the loss factor, tan (*δ*), which is an indicator of internal energy dissipation [198].

Within the framework of dynamic mechanical analysis, the loss factor is expressed by the following relation:

(5) $$tan\;\delta =\frac{E\prime \prime }{E\prime },$$

where *E*″ is the loss modulus and *E*′ is the storage modulus [198].

For a lightly damped system, the following approximate relationship may be used between the structural loss factor, *η*, and the damping ratio, *ζ*:

(6) η≈2ζ.

This relationship is useful for comparing results obtained by different experimental methods [195].

Vibration-damping evaluation tests can also be used to determine other quantities of engineering interest, such as natural frequencies, half-power bandwidth, transmissibility functions, maximum amplitude at resonance, equivalent modulus of elasticity, dynamic stiffness, and the fraction of energy dissipated per cycle. In the case of dynamic mechanical analysis, characteristic temperatures associated with the viscoelastic transitions of the polymer matrix are also considered [195,198]. In general, the results may be represented either by an amplitude–frequency diagram, featuring a resonance peak around the natural frequency, *f_n_*, from whose width the damping ratio can be estimated, or by an amplitude–time diagram, in which the oscillation envelope decreases exponentially, allowing the calculation of the logarithmic decrement [195,197]:

(7) $$\zeta =\frac{f_{2}-f_{1}}{{2f}_{n}},$$

where *f_n_* is the natural frequency and *f*_1_ and *f*_2_ are the frequencies corresponding to the half-power level of the resonance peak.

In the case of damped free oscillations, the logarithmic decrement is determined using the following equation:

(8) $$\zeta =ln(\frac{x_{n}}{x_{n+1}}),$$

where *x*_n_ and *x_n_*_+1_ represent two successive amplitudes of the same response [195,197].

The reviewed literature highlights the absence of a clear general trend regarding the influence of FDM process parameters on damping capacity, a fact that may be explained, at least in part, by the lack of uniformity in the indicators used. Some studies use the modal damping ratio ζ, or the loss factor, tan (δ), whereas others assess damping behavior indirectly through the resonance frequency, transmissibility function, or the response to impact and shock over time [195,197].

For this reason, apparently contradictory results may in fact reflect distinct physical mechanisms: an increase in porosity and in the density of interlayer interfaces promote dissipation through internal friction and may increase the modal damping ratio, whereas a high infill density may improve impact energy absorption and the behavior under severe dynamic loading. This latter situation is outlined in a previous study, in which PLA specimens tested by the rebound height method showed that infill percentage was the dominant factor, its increase leading to more efficient shock absorption [198,199].

In this section, the parameter extrusion height was not included as an independent variable, since the literature does not use it consistently in the same manner as layer height. In most of the studies analyzed, the geometric quantity controlled along the vertical deposition direction is layer height, whereas extrusion height appears only sporadically. Therefore, the analysis was limited to the parameters explicitly and consistently reported in the reviewed works, namely: infill percentage, printing speed, layer height, and nozzle temperature.

**Figure 18:**
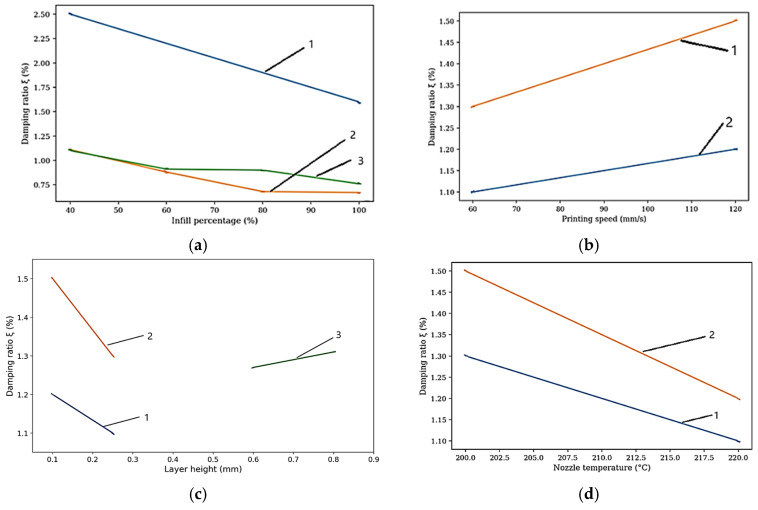
Vibration-damping ratio as influenced by: (**a**) infill density (1—[195]—ABS, 2—K [197]—ABS, 3—[197]—ABS), (**b**) printing speed (1—[196], series 1—PLA, 2—[196], series 2—PLA), (**c**) layer height (1—[196], series 1—PLA, 2—[196], series 2—PLA, 3—[197]—ABS), and (**d**) nozzle temperature (1—[196], series 1—PLA, 2—[196], series 2—PLA).

The values of the damping ratio ζ, extracted from the analyzed studies, are summarized in Table 8, and the corresponding trends are illustrated in Figure 18a–d. The table allows the direct comparison of numerical data from different studies, while the graphical representations highlight the direction and relative magnitude of the influence of each process parameter on vibration-damping capacity.

Based on the values presented in Table 8, the damping ratio, ζ, is sensitive to variations in process parameters; however, the magnitude and consistency of this influence differ both among the parameters themselves and across the studies analyzed.

In the case of infill percentage, Figure 18a shows that, in most situations, an increase in infill percentage is accompanied by a decrease in the damping ratio. In Series 1, for example, the value of ζ decreases from 2.50% to 1.60% as the level of infill increases from 40% to 100%, a trend that is also observed in the other data sets.

This may be explained by the fact that denser and stiffer structures limit local microdeformations and the internal friction mechanisms responsible for energy dissipation. However, in the 60–80% range, some deviations from a linear variation appear, suggesting the influence of other factors, such as internal topology, filament orientation, or the quality of interlayer bonding.

By contrast, for printing speed, the data show an opposite trend: an increase in printing speed is accompanied by an increase in the damping ratio, ζ, as can be observed in Figure 18b. In the first series, ζ increases from 1.10% to 1.20%, while in the second series, the increase is from 1.30% to 1.50%, for a speed variation between 60 mm/s and 120 mm/s. The probable explanation is that higher speeds reduce the time available for heat transfer and for the formation of optimal cohesion between layers, thereby favoring the occurrence of weakly consolidated interfaces and local inhomogeneities. Thus, a higher value of ζ does not necessarily indicate superior structural performance but may instead signal lower internal cohesion.

In the case of layer height, the influence is less uniform. For two of the data series, in the range of 0.10–0.25 mm, an increase in layer height is accompanied by a decrease in the damping ratio, *ζ*, from 1.20% to 1.10% and from 1.50% to 1.30%, respectively, a trend also illustrated in Figure 18c. However, another data series, corresponding to the 0.6–0.8 mm range, indicates a slight increase in *ζ*, from 1.27% to 1.31%, suggesting that the relationship between layer height and damping ratio is not monotonic and depends on the experimental context. Therefore, layer height influences vibration damping, but its effect cannot be regarded as univocal.

Concerning the nozzle temperature, the analyzed data indicate that increasing the printing temperature is accompanied by a decrease in the damping ratio, ζ, a trend also illustrated by the curves in Figure 18d. In the first series, the value of ζ decreases from 1.30% to 1.10% when the temperature increases from 200 °C to 220 °C, while in the second series, the decrease is from 1.50% to 1.20% over the same temperature interval.

Overall, FDM parameters influence damping capacity by modifying the internal architecture, structural homogeneity, and the quality of interlayer bonding. Parameters that promote more compact structures, such as high infill density and elevated nozzle temperature, tend to reduce the value of ζ, whereas parameters that accentuate internal inhomogeneity, such as high printing speed, tend to increase it. Layer height has a more complex effect, dependent on the variation range and experimental conditions. Thus, the values of ζ may be regarded not only as indicators of the dynamic response but also as indirect descriptors of the structural quality of the parts.

The literature also mentions other relevant factors, such as build orientation, infill pattern, and nozzle diameter, which influence vibration damping by modifying structural anisotropy, void distribution, and the degree of consolidation of interlayer interfaces [154,155,169,195,196].

## 15. FEM Simulation of FDM Process Conditions’ Effect on Polymer Mechanical Properties

The finite element method (FEM) tends to constitute a well-established numerical framework for solving boundary value problems governed by partial differential equations in solid mechanics, heat transfer, and coupled multi-physics phenomena. It rests on the discretization of a continuous domain into interconnected subdomains within which governing field variables are approximated through interpolation functions and equilibrium equations [200]. When applied to FDM, where layer-induced anisotropy, interbead voids, and temperature-dependent viscoelastic behavior render analytical solutions impractical, the typical FEM procedure encompasses geometry definition incorporating mesostructural features, constitutive model selection, mesh generation, boundary condition application replicating standardized mechanical tests, and numerical solution of the resulting algebraic system [201]. It also may contain advanced implementations, coupling thermal history simulations of the deposition process with structural analyses to embed residual stresses and bonding quality into the mechanical response [202]. Software platforms such as ANSYS, Abaqus, COMSOL Multiphysics, and MSC Marc are widely used by professionals, offering robust solvers, advanced material modeling capabilities that include user-defined subroutines for customized constitutive laws, and scripting interfaces that facilitate systematic parametric studies of process parameters [203]. The principal advantage of these environments lies in granting access to local stress and strain fields that are experimentally inaccessible, enabling virtual reproduction of standardized tests, and supporting process parameter optimization while significantly reducing the need for physical experimentation [204].

Regarding the phenomena of element birth and death, one must also take into account the residual stress and warping that may occur. In this regard, the adaptive octree mesh strategy of Gamdha et al. bridges G-code toolpaths and voxelized geometries in a scalable simulation framework, highlighting the interaction between transient thermal fields and residual stress development and identifying print speed, extrusion rate, and infill sparsity as numerically examinable parameters governing mechanical performance [205]. A more closely related approach is adopted by Ali et al. using ANSYS APDL combined with a MATLAB-based implicit backward-time central-space scheme, where computational findings reveal that a 0.1 mm layer height promotes thermal uniformity and reduces stress concentrations, while a 0.15 mm layer produces peak stresses of approximately 12 MPa due to increased thermal mass, thus correlating directly with warping defects observed in manufactured parts [206].

If crystallization kinetics, infill geometry, and process parameter effects are to be observed, a more refined treatment of the process–structure relationship is achieved by incorporating crystallization kinetics directly into the thermal finite element framework. Within a COMSOL environment, Samy et al. implement progressive element activation alongside a generalized Maxwell thermo-viscoelastic constitutive description and Nakamura crystallization kinetics. Parametric results demonstrate that reducing layer thickness from 0.5 mm to 0.1 mm yields a nearly 89% reduction in macroscopic warpage, while a transition from zig-zag to line raster reduces internal stress concentrations [207]. For short-carbon-fiber-reinforced PEEK, Qiu et al. incorporate dual-Avrami non-isothermal crystallization kinetics into an eight-node hexahedral element birth-and-death model that simulates a dynamic moving heat source integrating both nozzle temperature and an auxiliary laser power density. Their simulations demonstrate that maintaining ambient temperatures between 75 °C and 110 °C and laser power between 2 W and 3 W is enough for a boost that averages relative crystallinity by 60% to 82% compared to conventional rapid-cooling processes, while excessive laser densities are identified as capable of causing peak temperatures to exceed the decomposition threshold of PEEK [208]. The influence of infill pattern geometry has been examined using explicit mesostructural models, where Dezaki et al. use quadratic tetrahedral elements within Abaqus on concentric, grid, triangle, and zig-zag patterns to demonstrate that mechanical performance is fundamentally governed by the stress–strain behavior of the infill geometry rather than bulk material properties. Sala et al. show that, with linear elastic and van der Waals hyperelastic models, when increasing the nozzle diameter from 0.4 mm to 0.8 mm, it may elevate the compressive modulus and, at the same time, may help in identifying hexagonal patterns as mechanically inferior due to the absence of vertical supports. Bachhav et al. predict with ANSYS FEA that triangular infill at 70% density yields flexural stress of 38.82 MPa against 33.15 MPa for rectangular infill at the same density, also demonstrating the trade-off between material density and ductility [209,210,211].

With respect to the constitutive model selection, users may take into account the isotropic, elastoplastic, and hyperelastic formulations. The research of Pastor-Artigues et al. shows, through ANSYS simulations employing 20-node Solid 186 elements with geometric non-linearity, that printed PLA requires a bimodal constitutive framework rather than isotropic constants. Their simulation results are highly sensitive to specimen form and slenderness ratio, where excess material deposited at the corners of prismatic shapes creates a deterministic relationship between the FDM process path and final mechanical performance [212]. In a study of PETG under uniaxial compression, Mercado-Colmenero et al. find that the material can be effectively treated as isotropic in the elastic range in Ansys Mechanical, provided that directionally specific compression moduli derived from X, Y, and Z printing orientations are used, achieving displacement prediction errors between 2.80% and 3.98%. Their study explicitly recognizes the fact that the onset of plasticization introduces significant anisotropy requiring more advanced numerical treatment [213]. For materials subject to large deformations, hyperelastic formulations are employed by Gallup et al. and Bhuiyan et al.: the former use a third-order Mooney–Rivlin model for thermoplastic polyurethane in ANSYS, demonstrating that process-induced voids and layer adhesion directly govern material integrity and that differentiating shell from infill regions is critical for predictive accuracy, while the latter use neo-Hookean, Mooney–Rivlin, and Yeoh models combined with an inverse Levenberg–Marquardt algorithm on ABS to show that average Young’s modulus exhibits an exponential escalation with increasing infill density [214,215]. When corroborating, evidence is provided by Li and Zhu et al., whose simulations show that the equivalent von Mises stress reaches a stability plateau of approximately 50 MPa during sustained plastic flow, mirroring experimentally observed ductile failure, with a 10% increase in tensile strength over traditional manufacturing benchmarks attributed to molecular chain alignment along the deposition path [216].

If they are to observe internal geometry or infill configuration, researchers may prioritize them by means of mesostructural modeling. At the specimen level, Perera et al. use tetrahedral elements on PLA specimens to identify stress concentration zones at the interfaces between solid exterior walls and internal hollow regions as primary crack initiation sites, thus showing that circular versus rectangular cavity designs significantly alter load-bearing capacity and specific energy absorption. From a different perspective, Żur et al. use a virtualized compression test on specimens with a 1 mm outer PLA layer encapsulating various TPU infill strategies to derive a stiffness coefficient that validates material-saving V-shaped patterns against full-density cores [217,218].

At the mesostructural level, Khosravani et al. use C3D20R twenty-node quadratic brick elements with an elastoplastic constitutive model derived from experimental tensile tests on carbon-fiber-reinforced ABS in a cantilever configuration. They have shown by means of actively yielding parameters that smaller mesostructural cells facilitate a more uniform stress flow, thereby delaying yielding and enhancing maximum reaction force before failure compared to larger cells that concentrate stresses in isolated regions. Instead, Dezaki et al. map von Mises stress levels and displacement vectors across diverse PLA infill patterns under a constant 1000 N load, finding that grid and honeycomb configurations demonstrate exceptional structural resilience relative to their reduced mass, whereas rectilinear and wiggle designs sustain significantly higher stress and displacement [219,220]. At the filament-related geometry level, Hachimi et al. introduce a corrected virtual section accounting for the ovalization and flattening of deposited filaments calibrated through a Box–Behnken experimental design. They have achieved predictive accuracy exceeding 95% in the elastic zone and 90% in the elastoplastic zone for raster orientations of 0, 45, and 90 degrees. In turn, Sestini et al. and Alafaghani et al. model individual filaments as interconnected cylinders with defined welding zones, thus showing through sensitivity analyses that even marginal variations in layer spacing and filament diameter produce non-linear and substantial fluctuations in the predicted elastic modulus [221,222,223]. Homogenization-based approaches are pursued by Nasirov et al., who apply periodic boundary conditions through Lagrangian multipliers to a three-scale representative volume element framework to derive homogenized stiffness tensors. The procedure captures how meso-scale lattice structures interact with micro-scale filament properties. Differently, Rafiee et al. integrate finite element results from individual bead RVEs into classical lamination theory to predict Young’s modulus across rectilinear (0, 90) and (5, −45) raster orientations, therefore confirming higher stiffness for the (0, 90) configuration, thus demonstrating the efficacy of these tools in bypassing trial-and-error approaches in additive manufacturing [224,225].

Other aspects that are in the focus of this review are the ones related to topology optimization with process-induced limitations and specimen geometry corrections. The work of Zhang and Bai et al. explicitly integrates manufacturing constraints by evaluating overhang angles of individual struts against a 45° threshold within a hexahedral mesh topology optimization framework. They have shown by means of benchmarking on a Messerschmitt-Bolkow-Blohm beam model that lattice parameter distribution outperforms conventional cubic and body-centered cubic designs in structural compliance [226]. Complementing this, Zhang and Li et al. have integrated an algorithm by means of FEA in order to map stress fields and enable local densification or diameter enlargement in areas susceptible to premature failure. Their simulations of smooth transitions at lattice edges demonstrate predictive prevention of fractures caused by non-nodal connections or abrupt diameter changes [227]. Regarding simulation limitations arising from process-induced geometry, Wendt et al. use a mesh of over two million triangular elements to identify that the convergence of filament ends in V-shaped patterns at width transitions in standard dumbbell specimens and creates stress accumulations outside the gauge length and, on this basis, they propose and later validate an alternative rectangular geometry with reinforced grip areas. Thölking et al. similarly identify in an Abaqus laminate model with shell elements that, while raster orientation changes are predicted with high precision, upscaling of geometry leads to significant deviations between simulation and experiment, with a 20% discrepancy in curvature between parts produced by identical printers using the same G-code, underscoring the necessity of printer-specific model calibration [228,229].

The simulation of fracture-related phenomena in FDM-produced polymeric parts has been pursued at complementary scales of analysis. At the macro-scale, Ahmadi et al. demonstrate that FEM applied to specimens with intricate notch geometries constitutes a critical predictive tool for identifying stress concentration zones that precede structural failure. The study presents simulated distributions corroborated with experimental thermographic data, as it aims at revealing how raster orientation and layer adhesion exacerbate critical zones [230]. With a more explicit propagation model, Taoufik et al. implement the extended finite element method (XFEM) within Abaqus using C3D8R elements to address crack initiation and propagation in the discrete layer-by-layer architecture without constant remeshing. Their method captures the transition from stable to unstable crack growth as a function of the specimen’s life fraction, thus demonstrating that manufacturing conditions directly govern the stress field at the notch tip. By doing this, they also provide a mechanical-type explanation for the reduced fracture resistance of printed polymers relative to their bulk counterparts [231]. At the micro- and macro-scale, Monaldo et al. treat the macro-scale structure as a laminate assembly derived from a periodic unit cell capturing pseudo-elliptical filament cross-sections and interstitial holes. They incorporate a cohesive damage interface model for mode I, mode II, and mixed-mode fracture alongside an elastoplastic von Mises yield criterion for the bulk filaments. They achieve computational efficiency enhanced through the piecewise uniform transformation field analysis (PWUTFA) technique that approximates inelastic fields as piecewise constant functions across the unit cell [232]. Analogously, Polyzos et al. move beyond idealized geometric assumptions by extracting real fiber geometries via scanning electron microscopy and implementing them in reduced-integration tetrahedral RVEs with periodic boundary conditions, revealing that idealized models systematically underestimate the transverse elastic modulus and shear moduli by failing to account for the increased surface interaction and stress concentration zones inherent in the printed state [233].

Another group of researchers addresses multi-scale coupling between thermal history and mechanical deformation through finite element frameworks that account for the time- and temperature-dependent behavior of the printed polymer. The study of Smetannikov et al. uses the birth-and-death elements technique within ANSYS alongside a thermo-viscoelastic Prony series constitutive model. They have introduced relaxation parameters and the Williams–Landel–Ferry shift function calibrated from dynamic mechanical analysis data instead of general literature values. Their study shows that non-synchronous solidification of successive layers induces incompatible deformations, generating the characteristic residual warping of FDM parts and providing a significant contribution to the understanding of process–structure relationships [234]. Employing a related approach, Kostopoulos and Stamoulis et al. construct a global stiffness matrix with thermal force vectors based on the material’s coefficient of thermal expansion. They assign varying CTE values to distinct layers through a differential shrinking strategy in order to replicate the bending action resulting from specific FDM parameters. The Taguchi design of experiments (DoE) validation identifies layer height and activation temperature as the most significant governing factors. Extending this layer-resolved strategy, Kostopoulos and Georgantzinos et al. assign unique depth-dependent CTE coefficients to each layer within a six-layer mesh configuration, achieving submillimeter precision in predicting chord length, arc height, and internal arc length through integration of FEM with Taguchi experimental design and regression analysis. By doing this, they confirm the dominance of printing speed and layer height in governing the material’s internal thermal history and stress distribution [235,236].

Also, this section addresses the numerical investigation of melt flow within the FDM liquefier and nozzle assembly that has been pursued through finite element formulations. The steady-state laminar flow model of Gobena et al. applies a fine tetrahedral meshing strategy with double-precision iterations to the liquefier and nozzle geometry of PEEK. By incorporating the Cross-WLF viscosity model, the study quantifies how non-Newtonian flow and phase transitions respond to the shear rates and thermal exposure encountered during FDM operation. The computational data show that pressure drop is inversely proportional to nozzle diameter, with a reduction from 0.4 mm to 0.2 mm, thus elevating extrusion pressure, while temperature distribution maps reveal a terraced cooling gradient, which indicates that low thermal conductivity requires a precise balance of nozzle temperature and feeding speed to ensure adequate fluidity for robust interlayer fusion. These thermal-fluid simulations get mechanical validation in the finding that elevating printing temperature from 380 °C to 420 °C leads to an ultimate tensile strength of 120 MPa and improved impact strength due to enhanced molecular mobility and reduced porosity [237]. Moving beyond spherical particle fusion models, Li and Lou et al. implement a transient updated Lagrangian finite element formulation treating the molten polymer as an incompressible Newtonian fluid undergoing large deformations. A systematic variation of fiber volume fraction, shape, and eccentricity shows that embedded fibers actively hinder the flow of the molten thermoplastic, with insufficient neck growth at high fiber volume fractions. The methodology confirms that the internal bond strength is highly sensitive to the spatial distribution of the fiber phase within the thermoplastic matrix [238].

## 16. Mathematical Modeling and Optimization of FDM Process Conditions

In principle, the methods for mathematically modeling the influence exerted by input factors in the FDM process on the mechanical properties of polymeric materials aim to establish models that allow for formulating predictions regarding the values of the quantities defining the mechanical properties when the values of the input factors in the process are known. After establishing mathematical models of the nature mentioned above, the problem of optimizing the respective process can be formulated. In this case, the issue is identifying those values or combinations of values of the input factors in the FDM process such that it becomes possible to obtain the most suitable values for the quantities corresponding to one or more of the mechanical properties of the polymeric materials.

An analysis of the methods for mathematical modeling and optimization of the values of certain output parameters that define the mechanical properties of polymeric materials in parts manufactured via FDM by acting on the values of the input factors in the FDM process highlights the use of the following categories of methods:

1. *Statistical methods* for modeling and optimization, in which the results of experimental tests are used to generate mathematical models that will allow the creation of illustrative graphical representations. Such methods include: experimental design method [107,239], central composite design [103,104], response surface method [34,103,104,240,241,242,243,244], ANOVA method [103,104,107,241,243,244,245,246,247], Taguchi method [239,244,245,246,247,248,249,250], bacterial foraging technique or bacteria foraging optimization algorithm [103]; gray relational analysis [248,251], non-dominated sorting genetic algorithm II [239,242], differential evolution method [252], group method of data handling (GMDH) [252], multi-objective optimization [251,253], dragonfly algorithm [253], antlion algorithm [253], grey wolf algorithm [253], multi-verse algorithm [253], multi-objective dragonfly [253], fuzzy logic [240], dynamic mechanical analysis [107];
2. *Methods based essentially on creating graphical representations* that allow for a complete and suggestive illustration of both the input factors in the FDM process and the influence these factors exert on the values of the quantities defining the mechanical properties of polymeric materials. Such methods include, for example, the fishbone diagram method [254], Pareto-optimal solutions method [239], strength Pareto evolutionary algorithm (SPEA-II) [253], and the finite element method [65,100,238,239]. Some of the mentioned statistical, modeling, and optimization methods allow for, or are even finalized by, the development of graphical representations intended, among other things, to provide suggestive information regarding the influence exerted by the input factors in the FDM process on the values of certain quantities characterizing the mechanical properties of polymeric materials. In this regard, there are methods that consider input factors and interactions between them [107], methods for developing empirical mathematical models, the response surface method, the Taguchi method, response surface plots for determining optimal values of process parameters [103,104], the finite element method [65,100,255,256], etc.

A method of finalizing research aimed at modeling specific aspects or results of using the FDM process was *that of processing the results of experimental tests, leading to the proposal of empirical mathematical models*. The importance of such models stems from the possibility of evaluating, directly (for example, in the case of empirical mathematical models such as power functions or first-degree polynomials) or through graphical representations developed by considering the empirical mathematical models, the intensity of the influence exerted by certain input factors in the FDM process on the values of the mechanical properties of polymeric materials. For example, by analyzing the values of the coefficients in the case of mathematical models such as power functions or first-degree polynomials, observations can be made regarding the factors that have a determining influence on the values corresponding to certain mechanical properties and, respectively, the intensity of the influence exerted by those factors can be assessed.

A few examples of such second-degree polynomial mathematical models are those proposed by Panda et al. [103]:

- For the tensile strength *TS*:

*TS* = 13.5625 + 0.7156·*LT* − 1.3123·*O* + 0.9760·*RA* + 0.5183·*AG* + 1.1671·*LT*^2^ − 1.3014·*O*^2^ − 0.4363·*LT*·*O* + 0.4364·*LT*·*RW* − 0.436·*LT*·*AG* + 0.4364·*O*·*RA* + 0.4898·*O*·*AG* − 0.5389·*RA*·*RW* + 0.5389·*RA*·*AG* − 0.5389·*RW*·*AG*; (9)

- For the flexural strength *FS*:

*FS* = 29.9178 + 0.8719·*LT* − 4.8741·*O* + 2.4251·*RA* − 0.9096·*RW* + 1.6626·*AG* − 1.7199·*LT*·*RA* + 1.7412·*LT*·*RW* − 1.1275·*LT*·*AG* + 1.0621·*O*·*AG* + 1.0621·*RA*·*AG* + 1.0408·*RW*·*AG*; (10)

- For the impact strength *IS*:

*IS* = 0.401992 + 0.034198·*LT* + 0.008356·*O* + 0.013673·*RA* + 0.021383·*LT*^2^ + 0.008077·*O*·*RW*, (11)

In these equations, the symbols have the following meanings: *LT*—layer thickness, *O*—orientation, *RA*—raster angle, *RW*—raster width, *AG*—air gap.

Other examples of empirical mathematical models are those proposed by Mohamed et al. [107] regarding certain mechanical properties specific to the dynamic behavior of polymeric materials in parts manufactured via FDM:

- For the storage modulus *SM*:

*SM* = 838.87 − 1929.66·*LT* − 1324.27·*AG* − 2.95·*RA* + 803.24·*RW* + 12.442·*NC* − 1294.27·*LT*·*AG* + 11.25·*LT*·*RA* − 3867.97·*LT*·*RW* + 135.81·*AG*·*NC*; (12)

- For the loss modulus *LM*:

*LM* = 138.77 + 31.10·*LT* − 128.96·*AG* − 0.432·*RA* + 0.238·*NC* − 324.94·*LT*·*AG* + 1.748·*LT*·*RA* + 15.685·*AG*·*NC*; (13)

- For the mechanical damping *MD*:

*MD* = 0.848 + 0.0125·*LT* − 0.234·*AG* − 3.926·10^−4^·*RA* + 3.005·10^−4^·*BO* + 0.228·*RW* − 0.0125·*NC* − 0.63615·*LT*·*AG* + 1.802·10^−3^·*LT*·*RA* − 2.293·10^−3^·*LT*·*BO* + 0.0855·*LT*·*NC* + 0.0392·*AG*·*NC*, (14)

where *LT* is the layer thickness, *AG*—air gap, *RA*—raster angle, *BO*—build orientation, *RW*—road width, and *NC*—number of contours.

It is worth noting that mathematical models from the category of those mentioned above also provide useful information regarding the influence of interactions between input factors in the FDM process on the characteristic values of some mechanical properties of polymeric materials. Other observations regarding the influence exerted by interactions between input factors in the FDM process have also been highlighted in the previous sections of this work.

The main software used for modeling and optimizing the influence exerted by input factors in the FDM process on the mechanical properties of materials in parts manufactured through this process were: Minitab/Design-Expert (for modeling using the response surface method, the Taguchi method, and for design of experiments methods), Python (for obtaining additional or advanced information), MATLAB (for modeling using the response surface method and for undertaking optimization approaches), ANSYS/Abaqus/SolidWorks Simulation (for obtaining information regarding, for example, the distribution of internal stresses using the finite element method or another method), etc.

**Figure 19:**
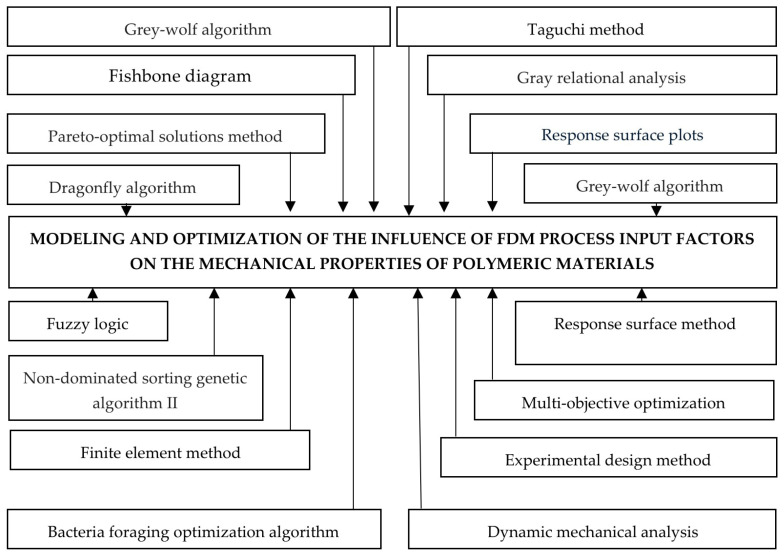
Methods used for modeling and optimizing the influence exerted by input factors in the FDM process on the mechanical properties of polymeric materials. (Grey-wolf algorithm [253], Fishbone diagram [254], Pareto-optional solutions method [239], Dragonfly algorithm [253], Taguchi method [244,245,246,247,248,249,250], Grey relational analysis [248,251], Response surface plots [103,104], Grey-wolf algorithm [253], Fuzzy logic [240], Non-dominated sorting genetic algorithm II [239,242], Finite element method [65,100,255,256], Bacteria foraging optimization algorithm [103], Response surface method [34,103,104,240,241,242,243,244], Multi-objective optimization [251,253], Experimental design method [107,239], Dynamic mechanical analysis [107]).

Some of the methods used for modeling and optimizing the influence exerted by input factors in the FDM process on the mechanical properties of polymeric materials in parts manufactured via an FDM process can be observed in Figure 19.

Some limitations of the research conducted to date derive from the still small number of influencing factors considered when addressing the issue of modeling the influence of these factors on the mechanical properties of polymeric materials. While, in most research, the influence of typically 3–5 input factors was analyzed, the maximum number of such factors was 11 [257]. In a review article, Cojocaru et al. showed that, by 2022, 16 input factors had been considered for analyzing the influence exerted by these factors on the mechanical properties of polylactic acid in parts manufactured via FDM [31]. Before effectively proceeding to establish the input factor values that ensure obtaining optimal values for the quantities corresponding to the mechanical properties of polymeric materials, it may be necessary to select those factors presumed to exert an important influence on the values of the output parameters of the FDM process. For this purpose, it is possible to use an optimal input factor selection method, employing a decision-making optimization method; one of these methods is the rank correlation method [258].

## 17. Conclusions

The research presented in this paper aimed at the systematic analysis of how the conditions of the fused FDM process influence the mechanical properties of polymeric materials. These materials, defined by their versatile macromolecular structure, are processed through the FDM process by the layered extrusion of thermoplastic filaments, a method that allows for obtaining controlled variations in mechanical performance. The analysis highlighted that properties essential for practice, such as tensile, compressive, flexural, and impact strength, can be significantly optimized through the rigorous adjustment of input factors, the choice of composite materials, or the application of thermal post-processing treatments.

Among the main input factors analyzed, infill density, part orientation, and thermal parameters proved to be determinant, with infill density generally exerting the greatest influence on the structural integrity of 3D-printed parts. The research hypotheses were confirmed by the specialized literature. It was found that the mechanical performance critically depends on the quality of the interlayer bonds and the deposition paths, identifying strength increases of up to 25% under optimal post-processing conditions. For modeling these complex interactions, current research has migrated from simple experimental analyses towards advanced optimization methods, such as the finite element method, genetic algorithms, and machine learning techniques.

It is noteworthy that researchers have been involved in numerical and mathematical modeling of the studied process as a tool to highlight the influence of different factors on the values that characterize the mechanical properties of polymeric materials.

The evolution of studies in the field reflects a clear trend of moving from monofactorial analyses towards complex multifactorial approaches and the use of high-performance materials, as evidenced by the spectacular increase in interest in PLA over the last decade. However, the current study highlights certain limitations, particularly the focus on standardized test specimens at the expense of functional components with complex geometries. Therefore, future research directions must concentrate on validating predictive models under real use conditions and on exploring more deeply the synergistic interactions between FDM process parameters to ensure an efficient transition towards high-precision industrial applications.

## Figures and Tables

**Table 1:** The evolution of scientific and technical knowledge regarding the influence of FDM process conditions on the mechanical properties of polymeric materials.

Year	Identified Information
2000	Results of research regarding the influence of layer orientation on certain mechanical properties of materials in parts manufactured via FDM were published by Es-Said et al. [97].
2001	Montero et al. addressed the issue of characterizing materials in parts manufactured via FDM using designed experiments [98].
2002	Ahn et al. published results of research on the anisotropy of ABS incorporated in parts manufactured via FDM [99].
2003	Rodríguez et al. used the asymptotic homogenization theory and the finite element method to model the stiffness and strength of acrylonitrile butadiene styrene [100].
2003	Ahn et al. published an article regarding the modeling of the anisotropic tensile behavior of materials in parts manufactured via FDM [101].
2003	Bellini and Güçeri addressed the issue of the influence of certain FDM process parameters on mechanical properties [102].
2009	Panda et al. published results of research aimed at optimizing the FDM process from the perspective of certain mechanical properties [103].
2010	Sood et al. published results of research regarding the mechanical properties of parts manufactured via FDM [104].
2010	Masood et al. referred to the tensile strength of polycarbonate in parts processed via FDM [105].
2015	Brensons et al. published results of research regarding the optimization of the FDM process to ensure high tensile strength [86].
2016	Christiyan et al. referred to the results of flexural tests [106].
2016	Mohamed et al. published a paper in which aspects regarding the influence of FDM process parameters on dynamic mechanical properties (storage modulus, loss modulus, mechanical damping) were addressed [107].
2017	A new version of ASTM D790 was proposed, which takes the flexural testing method of polymeric materials into greater consideration. A final version of this standard appeared in the year 2025 [53].
2018	Results of systematic research regarding the influence of FDM process conditions on flexural strength were published by Gebisa et al. [108].
2018	Balderrama-Armendariz et al. published results of experimental torsion tests performed on ABS M30 specimens [109].
2018	Popescu et al. published a review article in which they analyzed the influence of input factors in the FDM process on the mechanical properties of materials [32].
2019	Patterson et al. published one of the first articles addressing the issue of the influence of FDM process conditions on impact strength [110].
2019	Syrlybayev et al. published a critical review regarding the optimization of strength properties of parts manufactured via FDM [34].
2021	Zisopol et al. communicated results of research regarding the influence of bed temperature on the hardness of the material of parts manufactured via FDM [111].
2022	Popa et al. communicated results of research regarding the impact strength of PLA specimens [112].
2022	Gao et al. published the results of an extensive analysis regarding the influence of input factors in the FDM process on the mechanical properties of materials incorporated in parts manufactured via FDM [33].
2024	Results of research regarding the influence of input factors in the FDM process on the tensile strength of PLA were communicated by Megersa et al. [113].
2025	Gajjar et al. published the results of experimental research regarding the influence of the most important input factors in the FDM process on the mechanical properties of materials incorporated in parts manufactured via this process [114].
2025	Ramos et al. published an article in which they addressed the issue of the influence of layer orientation on the mechanical properties of polymeric materials in parts manufactured via FDM [115].

**Table 2:** Summary statistics of tensile strength for five pure thermoplastic materials processed via FDM.

Material	Investigated Variables	σ Range (MPa)	ε Range (%)	Variability	Standard	Data Points	Source
PLA	Infill density (40–100%), honeycomb, 0.2 mm layer, 210 °C	22.49–45.00	4.23–4.68	Low	ISO 527-2	20	[75]
PLA+CF	Infill density (40–100%), honeycomb, 0.2 mm layer, 225 °C	23.09–42.54	~4.0–4.5	Low–Mod.	ISO 527-2	20	[75]
PLA	Temperature (180–220 °C), speed (35–45 mm/s), 20% infill, ±45°	~28–48	Variable	Moderate	ASTM D638	12	[72]
PETG	Temperature (225–245 °C), speed (25–35 mm/s), 20% infill, ±45°	~22–38	Variable	Moderate	ASTM D638	12	[72]
ABS	Speed and raster angle variations, 100% infill, 235 °C	~32–36	40–90	Low	ASTM D638	9	[71]
PLA+	Raster angle (0°/0°, 0°/90°, 45°/−45°), fixed infill	N/R	Highest at 45°	Moderate	ASTM D638	9	[77]
PLA	Infill (25–100%), orientation (0°, 45°, 90°)	Increases with infill	Decreases	Moderate	ASTM D638	12	[121]
PEEK	Build orientation, path (L, W, T), 100% infill	37.45–>60	<5% to 96%	Very High	ISO 527	18	[79]

**Table 3:** Summary statistics of compression for two pure thermoplastic materials processed via FDM.

Material	Investigated Variables	Comp. Strength Range (MPa)	Variability	Standard	Data Points	Source
PLA	Infill (25–100%), hexagonal, 0.2 mm, 200 °C	25%: ~18; 100%: ~42	Low–Moderate	ASTM D695	12	[121]
PLA	Infill (20–80%), pattern (Hilbert, rectilinear, honeycomb, etc.), 0.2 mm	80% Hilbert: 121.35; honeycomb: 62.56	Moderate–High	ASTM D695	24	[122]
PLA	Layer config. (single, double, four), pattern (triangular, honeycomb, grid)	50%: ~15–25	Moderate	Custom	18	[124]
PETG+CF	Pattern (trihexagon, cubic, line), infill (40–80%), 230 °C	80% trihexagon: 39.16; 40% cubic: 11.52	Moderate–High	ASTM D695	13	[123]
PLA	Temperature (180–220 °C), speed (35–45 mm/s), 20% infill, ±45°	~15–30	Moderate	ASTM D695	12	[72]
PETG	Temperature (225–245 °C), speed (25–35 mm/s), 20% infill, ±45°	~12–25	Moderate	ASTM D695	12	[72]

**Table 4:** The influence of FDM process parameters on impact strength.

Main Influencing Factors	Parameter Value	Impact Resistance [kJ/m^2^]	References	Material
Infill [%]	20	14.8	[152]	PLA
	50	21.3	[152]	PLA
	80	27.4	[152]	PLA
	100	30.5	[152]	PLA
	40	34.66	[156]	PLA
	60	36.12	[156]	PLA
	80	37.01	[156]	PLA
	100	38.54	[156]	PLA
	20	16.9	[159]	PLA
	50	22.8	[159]	PLA
	100	32	[159]	PLA
Printing speed [mm/s]	30	19.7	[152]	PLA
	50	21.3	[152]	PLA
	70	18.5	[152]	PLA
	40	34.66	[156]	PLA
	60	36.91	[156]	PLA
	75	38.54	[156]	PLA
	40	17.4	[157]	PLA
	60	19.1	[157]	PLA
	80	16.8	[157]	PLA
Layer height [mm]	0.1	20.5	[152]	PLA
	0.2	21.3	[152]	PLA
	0.3	19.8	[152]	PLA
	0.1	18.3	[157]	PLA
	0.2	19.1	[157]	PLA
	0.3	17.6	[157]	PLA
	01	2.04	[158]	PLA
	0.2	2.52	[158]	PLA
	0.3	2.13	[158]	PLA
Extrusion width [mm]	0.35	20.1	[158]	PLA
	0.4	21.3	[152]	PLA
	0.45	20.4	[152]	PLA
	0.35	18.1	[157]	PLA
	0.4	19.1	[157]	PLA
	0.45	18.3	[157]	PLA
Nozzle temperature [°C]	190	18.6	[152]	PLA
	200	21.3	[152]	PLA
	210	23.1	[152]	PLA
	195	35.8	[156]	PLA
	205	38.54	[156]	PLA
	215	37.2	[156]	PLA
	200	18.5	[157]	PLA
	210	19.1	[157]	PLA
	220	20.4	[157]	PLA

**Table 5:** Values of the constants A and B from the researched sources.

Material	A	B	Bibliographic Source
Nylon	206	−0.039	[167]
PLA	1511.62	−0.366	[168]
ABS	164.28	−0.199	[169]
ABS	63.31	−0.204	[170]
ABS	167.26 395.67	−0.2782 −0.3831	[171]

**Table 6:** FDM parameters with influence on fatigue strength, for polymeric materials, depending on the type of fatigue testing.

	Main Influencing Factors								
	Material	Specimen Type	Deposition (Raster) Orientation	Infill Percentage	Printing Speed	Layer Height	Extrusion Height	Nozzle Temperature	Reference
Tension—tension pulsating	ABS	Dog bone	Zig-zag, 45 o	Not specified	Not specified	0.3048	0.1778 mm	320 o	[173]
	ABS	Dog bone	Not specified	Not specified	Not specified	Not specified	Not specified	Not specified	[174]
Tension—tension pulsating	PLA	Dog bone	45 o	Not specified	Not specified	Not specified	Not specified	Not specified	[175]
Tension—tension pulsating (cyclic)	Ultem 9085	Dog bone	Not specified	Not specified	Not specified	Not specified	Not specified	195 o	[176]
	ABS	Dog bone	45 o	Not specified	Not specified	Not specified	0.1 mm	Not specified	[177]
Fatigue in tension (tension—relaxation)	PU, PCU	Rectilinear	circular	100%	7 mm/s	Not specified	0.1 mm	205 o	[178]
	Nylon	Triangular	0 o	20%	Not specified	Not specified	0.1 mm	Not specified	[167]
	PLA	Dog bone	45 o 0 o	50% 75%	40 mm/s	Not specified	0.2 mm	215	[179]
Compression fatigue	PLA	Rectilinear	Circular	60%	Not specified	Not specified	0.4 mm	Not specified	[180]
Rotating bending	PLA	Honeycomb	Not specified	75%	25, 30, 35 mm/s insignificant	0.3 mm	0.5 mm	Not specified	[181]
	PLA	Rectilinear	horizontal	60%	30 mm/s	0.2 mm	1	170–240 o	[182]
	ABS	Honeycomb	Not specified	100%	30 mm/s	0.1 mm	0.5 mm	220–230 o	[183]
	ABS Nylon	Trihexagonal	Horizontal	20%	35 mm/s	0.15 mm	0.2 mm	220 o 210 o	[184]
	ABS PLA	Square	Horizontal and vertical	50%	60 mmm/s	0.15 mm	0.4 mm	245 o	[171]
	PLA with fiber	Circular	Grid	100%	40 m/s	0.2 mm	0.4 mm	215 o	[185]
	PLA	Circular	30 o	100%	20 m/s	0.2 mm	0.4 mm	215 o	[186]
	PLA	Rectilinear	0 o	100%	20 mm/s	0.3 mm	0.6 mm	2200	[168]
	ABS	Honeycomb	45 o	75%	35 m/s	0.2 mm	0.4 mm	230 o	[169]
Mechanical fracture	PLA	Compact	0/90 o	80%	80 mm/s	0.25 mm	0.5 mm	250 o	[187]
Vibration fatigue	PLA	Rectangular	45 o	100%	80 m/s	0.2 mm	0.4	220 o	[172]
Monotonic torsion tests	ABS	Zigzag		70%	40 m/s	0.15 mm	0.5 mm	220 o	[170]
Fatigue by bending	ABS	Rectangular	Horizontal	Not specified	Not specified	0.15 mm	0.8 mm	245 o	[188]

**Table 7:** Influence of input parameters on hardness and observations.

Input Factor	Effect on Hardness	Physical Explanation and Comments
Infill	Direct increase	Higher infill provides solid structural support beneath the top layer, reducing global deformation under the indenter [193].
Extrusion Temperature	Increase (up to an optimum)	High temperatures favor molecular diffusion at the layer interface, eliminating microvoids that reduce hardness [194].
Layer Thickness	Contradictory results	Some studies indicate higher hardness with thin layers due to compaction, others with thick layers due to thermal mass [193].
Printing Orientation	High anisotropy	Hardness is maximum when the indentation force is applied perpendicular to the layer deposition direction [193].
Fan Speed	Decrease (with rapid cooling)	Excessively rapid cooling of the deposited layers can lock the material into an amorphous state with low density and can limit the time necessary for optimal molecular fusion [194].

**Table 8:** The influence of selected input factors in the FDM process on vibration-damping capacity.

Main Influencing Factors	Parameter Value	Damping Ratio ζ [%]	References	Material
Infill [%]	40	2.50	[197]	ABS
	100	1.60	[197]	ABS
	40	1.11	[195]	ABS
	60	0.88	[195]	ABS
	80	0.68	[195]	ABS
	100	0.67	[195]	ABS
	40	1.10	[195]	ABS
	60	0.91	[195]	ABS
	80	0.90	[195]	ABS
	100	0.76	[195]	ABS
Printing speed [mm/s]	60	1.10	[196]	PLA
	120	1.20	[196]	PLA
	60	1.30	[196]	PLA
	120	1.50	[196]	PLA
Layer height [mm]	0.1	1.20	[196]	PLA
	0.25	1.10	[196]	PLA
	0.1	1.50	[196]	PLA
	0.25	1.30	[196]	PLA
	0.6	1.27	[195]	ABS
	0.8	1.31	[195]	ABS
	0.1	1.20	[196]	PLA
	0.25	1.10	[196]	PLA
	0.1	1.50	[196]	PLA
Nozzle temperature [°C]	200	1.30	[196]	PLA
	220	1.10	[196]	PLA
	200	1.50	[196]	PLA
	220	1.20	[196]	PLA
	200	1.30	[196]	PLA
	220	1.10	[196]	PLA

## Data Availability

No new data were created or analyzed in this study. Data sharing is not applicable to this article.
